# Integrating motif, DNA accessibility and gene expression data to build regulatory maps in an organism

**DOI:** 10.1093/nar/gkv195

**Published:** 2015-03-19

**Authors:** Charles Blatti, Majid Kazemian, Scot Wolfe, Michael Brodsky, Saurabh Sinha

**Affiliations:** 1 Department of Computer Science, University of Illinois, Urbana, IL 61801, USA; 2 National Heart Lung and Blood Institute, National Institutes of Health, Bethesda, MD 20892, USA; 3 Program in Gene Function and Expression, University of Massachusetts Medical School, Worcester, MA 01655, USA; 4 Department of Molecular Medicine, University of Massachusetts Medical School, Worcester, MA 01655, USA; 5 Department of Biochemistry and Molecular Pharmacology, University of Massachusetts Medical School, Worcester, MA 01655, USA; 6 Institute of Genomic Biology, University of Illinois, Urbana, IL 61801, USA

## Abstract

Characterization of cell type specific regulatory networks and elements is a major challenge in genomics, and emerging strategies frequently employ high-throughput genome-wide assays of transcription factor (TF) to DNA binding, histone modifications or chromatin state. However, these experiments remain too difficult/expensive for many laboratories to apply comprehensively to their system of interest. Here, we explore the potential of elucidating regulatory systems in varied cell types using computational techniques that rely on only data of gene expression, low-resolution chromatin accessibility, and TF–DNA binding specificities (‘motifs’). We show that static computational motif scans overlaid with chromatin accessibility data reasonably approximate experimentally measured TF–DNA binding. We demonstrate that predicted binding profiles and expression patterns of hundreds of TFs are sufficient to identify major regulators of ∼200 spatiotemporal expression domains in the *Drosophila* embryo. We are then able to learn reliable statistical models of enhancer activity for over 70 expression domains and apply those models to annotate domain specific enhancers genome-wide. Throughout this work, we apply our motif and accessibility based approach to comprehensively characterize the regulatory network of fruitfly embryonic development and show that the accuracy of our computational method compares favorably to approaches that rely on data from many experimental assays.

## INTRODUCTION

Describing gene expression and its regulation remains one of the grand challenges of biology today. Scientists generating gene expression measurements are eager to understand the regulatory mechanisms governing their observations both at the molecular level and at the organismal level. Specific aims of their enquiry typically include identifying transcription factors (TFs) that regulate gene expression in a cell type as well as locating and characterizing relevant enhancers responsible for specific regulatory activity, e.g. driving expression in a particular spatio-temporal pattern. A variety of genome-wide technologies have been developed to answer these types of questions. ChIP-seq assays are used to profile whole genome transcription factor (TF)–DNA binding and epigenomic states involving histone modifications, DNase-seq, FAIRE-seq, etc., are used to characterize DNA accessibility, and chromatin capture methods such as Hi-C identify physical interactions between different parts of the genome (1).

Combinations of these emerging technologies have allowed groups to study gene regulation more comprehensively than previously possible. However, for the overwhelming majority of biologists, especially those studying cell types or species that are not included in the ENCODE and modENCODE projects, many of these assays remain expensive and/or challenging (e.g. due to requirement of unrealistic quantities of sample) to apply to their system of choice. Computational methods that help uncover regulatory networks and elements of gene expression from sequence and limited experimental data are thus in demand and the subject of this study.

There has been significant investment in generating catalogs of genome-wide TF–DNA binding, via ChIP-chip and ChIP-seq assays, in various cell types. TF-ChIP assays have been successfully used by some groups (2–7) to characterize TF roles and predict enhancer activity. However, such assays require substantial numbers of cells and high-quality antibodies, making them difficult to scale to large numbers of TFs and cell types. On the other hand, various recent studies (8–12) have shown the feasibility of predicting cell type-specific TF–DNA binding profiles by utilizing experimentally characterized TF–DNA binding specificities (‘motifs’) and chromatin accessibility data. This ‘motif + accessibility’ strategy has a practical appeal: it produces TF–DNA binding predictions for hundreds of TFs by combining computational scoring of genomic sequence for TF motifs with a single accessibility assay to reflect cell type specific regulatory information.

Far more TFs have had their motifs characterized with *in vitro* assays than have had adapted for ChIP-seq analysis (13). For example, while about 60% of the nearly 1400 human TFs have motifs available today (14), <10% of human TFs in the ENCODE project (15) have ChIP data available in a limited number of cell types/lines. It is reasonable to expect that in the near future, most TFs in human and certain model organisms will have characterized motifs either from direct experimental assay or by imputation via homology. Initial work (16,17) demonstrates the possibility of using these motif collections to perform regulatory analysis on less studied organisms. In other words, from a practical point of view, a strategy for reconstructing regulatory relationships based on motif and accessibility data can have widespread impact if it has demonstrated predictive value.

In this paper, we present and comprehensively evaluate a method that is systematically designed for its ability to identify regulatory networks and elements that control gene expression in poorly characterized cell types using only TF motifs and limited experimental data. We confirm previous reports that motifs and accessibility data can be used to effectively predict TF–DNA binding potential genome-wide. We then show that (a) the predicted TF binding potentials can be used together with TF expression data to identify relevant TFs playing major roles in specific cell types, and (b) the relevant TFs thus identified can be used to predict enhancer activities. While the general predictive framework outlined above is intuitively appealing, we are not aware of work that tests its potential and limitations systematically and comprehensively.

There have been studies (8,9,11,12,18–22) exploring how well motifs and/or accessibility data can predict ChIP-based occupancy profiles, but those studies have not gone on to assess where these approaches stand vis-à-vis the ultimate goal of identifying relevant TFs and predicting enhancer activities. Several studies (2–4,6,7,23) have utilized cell type-specific ChIP data to characterize TFs involved in a transcriptional network, while others (24–27,28) have demonstrated motif based computational approaches to infer binding and then gene expression. These studies have typically been limited to very few, well characterized cell types and regulatory networks, where much prior knowledge exists in the form of relevant TFs, genetic knockdowns, validated enhancers, etc. In this work, we show that computational methods using motifs and accessibility can be successfully applied in a number of different cell types without requiring extensive prior knowledge.

There has been exciting progress recently in terms of identifying enhancers active in a cell type using chromatin state data (29) and in identifying the associated gene based on spatial organization maps of the chromatin (30). However, it remains challenging to determine the exact regulatory output of an enhancer — genes frequently have multi-faceted expression patterns and harbor multiple enhancers in their intergenic regions, each of which may correspond to some facet of the expression pattern. It has been suggested that dynamic chromatin states paint broad brushstrokes of the regulatory landscape, while transcription factors help set up more nuanced, cell type-specific expression programs (10,31). Thus, the emerging strategy of assigning enhancer driven expression based on chromatin states is expected to lead to ambiguities. In this work, we assess the extent to which this is the case, and to what extent the ambiguities in activity prediction may be reduced by utilizing additional information on TF expression and binding potentials.

We chose to perform this study in the context of embryonic development in *Drosophila melanogaster*, because of the relatively mature status of the data types involved. We found that experimentally characterized fruitfly TF motifs along with developmental stage-specific accessibility data can accurately predict ChIP-based TF–DNA binding profiles in those stages, as reported previously (9,12), and that the predictions are more accurate when combining motif scores from multiple *Drosophila* species. This analysis revealed motifs whose scores correlate very strongly with accessibility, consistent with recent studies (32), and also motifs that are strongly anti-correlated with accessibility, which to our knowledge is a novel observation.

We next evaluated whether our motif score profiles can identify putative regulators of sets of genes expressed in ∼200 distinct spatial expression domains in the early *Drosophila* embryo. Through systematic testing, we identified a strategy for identifying TFs associated with an expression domain that best agrees with data on the TF's expression in that domain. Using this strategy, we built a compendium of TF–domain associations involving 195 TFs and 88 expression domains, and made this data available through an easy-to-navigate online interface [http://veda.cs.uiuc.edu/B1H_GRN]. Analysis of the new compendium revealed TFs and expression domains with systematic biases for regulatory regions that are gene-proximal or distal. Importantly, we found this motif-based strategy for TF function assignment to be as accurate as an identical approach that uses ChIP data in place of motif scores.

Finally, we annotated candidate enhancers, defined as developmental stage-specific open chromatin regions, for the likely expression pattern they produce. This was done using a regression model that incorporated predicted TF binding, TF expression, and the results of our functional associations. Without using any prior knowledge to train our models, we were able to recover accurately enhancers for over half of the 40 separate expression domains with existing data that allows evaluations. In summary, we show how motifs, DNA accessibility and expression data may be integrated computationally to characterize gene regulatory networks, and find that this can be done often with comparable accuracy as with ChIP data.

## MATERIALS AND METHODS

### Creating scoring profiles

We created motif score profiles from a collection of motifs for 325 distinct fruitfly TFs that were characterized with the bacterial 1-hybrid (B1H) technology (33) and made these available through the Genome Surveyor interface [http://veda.cs.uiuc.edu/gs]. Our motif score profiles were calculated with the Stubb tool (34), which integrates any number of strong or weak predicted sites to estimate a score of TF–DNA binding for each 500 bp-long window in the genome with 50 bp shifts. We also computed multi-species averages of these motif profiles by using a phylogenetically weighted averaging (24) of motif scores from orthologous segments in 12 *Drosophila* genomes (additional details in Supplementary Methods SM1). To evaluate the accuracy of these computational profiles, we collected 69 ChIP data sets (Supplementary Table S1), representing 40 TFs during early development, from the ModENCODE consortium (35) and other studies (2,36–38) (SM2). The raw ChIP data was converted into averaged values for each of our 500 bp genomic windows by averaging the maximum read scores from each 50bp subsegment of the window. We transformed our motif scores into ‘motif + accessibility’ scores by integrating DNaseI-seq chromatin accessibility data from BDTNP (39) from five stages of embryonic development (5, 9, 10, 11 and 14). The raw accessibility data was also averaged for each of our 500 bp genomic windows and only the top 10% of windows within each developmental stage (18) were considered accessible and their original motif scores reported (SM3).

### Identifying TF–domain associations

We created a ‘gene expression atlas’ based on data from the Berkeley *Drosophila* Genome Project (BDGP) (40). This atlas comprises 7212 genes organized into 195 non-exclusive ‘expression domains’, i.e. tissue or cell types and developmental stage describing the gene's expression pattern. These domains span four developmental stages labelled ‘4–6’, ‘9–10’, ‘11–12’ and ‘13–16’ (SM5, Supplementary Table S5, and Figure S4). We predicted sets of genes potentially regulated by a TF (‘TF target set’) as genes whose regulatory regions had the strongest evidence of motif occurrence from our chromatin accessibility filtered multi-species motif scans (SM6 and Supplementary Note 2). The TF target set was tested for overlap with genes expressed in an expression domain, and a significant overlap was used as evidence of the TF's broad role in specifying the expression domain. We call such a statistical finding a ‘TF - domain association’. We utilized three different definitions of gene regulatory regions to use when predicting TF targets: 1 kb upstream (‘p1K’) or 5 kb upstream (‘p5K’) of the transcription start site, or a regulatory region that extends for up to 50 kb on either side of the gene unless terminated by insulator marks (‘IG’, see SM7). A TF target set was constructed by each of these three definitions of regulatory region, *P*-values of TF–domain associations were computed, and the region definition producing the lowest *P*-value was selected.

### Modelling enhancer activity

We defined our set of candidate enhancers as non-overlapping 500 bp windows that were in the top 10% of accessible windows in any of the four embryonic developmental stages and had comparable motif content to known REDfly enhancers (SM12). We used gene proximity, gene expression annotations, and accessibility profiles of enhancers to create a preliminary assignment of putative enhancers to specific genes and expression domains for training our enhancer models. For each expression domain, *D*, we created a model positive training set of up to 500 open regions that were within 5 kb of their neighboring gene annotated with *D*, accessible during the developmental stage of *D*, and did not overlap any REDfly enhancers. We then selected a matching number of negative examples (open regions whose neighboring genes are not annotated with *D*, accessible during the developmental stage of *D*, and did not overlap any REDfly enhancers). We used 75% of the data for training and left out the remaining 25% for evaluation of the model. For 40 expression domains with sufficient data, we additionally created ‘REDfly versus Open Regions’ (RFVO) evaluations which only use open regions overlapping the REDfly enhancers as positives examples for training and testing (SM13).

Our domain specific enhancer models were designed to capture relevant properties including motif and chromatin accessibility scores as well as TF expression levels and the TF–domain associations determined above. The activity-prediction model (henceforth called the ‘complete’ enhancer model) for a domain *D* can be described as:

\begin{equation*} y^r = \sum\limits_{m = 1}^{325} {\alpha _m Z_m^r S_m^D E_m^D R_m^D } + \sum\limits_{s = 1}^4 {\gamma _s A_s^r + \beta } \end{equation*}

where *y^r^* is the prediction indicating whether region *r* is in the positive set; *m* is one of the 325 motifs; *s* is one of the four developmental time points (stage 5, 9, 11 and 13); *α_m_, γ_s_* and *β* are the domain-specific parameters; $Z_m^r$ is the non-negative multi-species motif scores for region *r* for the *m*th motif; $S_m^D$ is the negative logarithm of the *P*-value of association between the expression domain *D* and the TF represented by the *m*th motif; $E_m^D$ indicates whether the TF related to the *m*th motif is expressed in *D* or in a related expression domain; $R_m^D$ is the ‘fragments per kilobase of exon per million fragments mapped’ (FPKM) reported from (41) for the TF related to the *m*th motif in the developmental stage related to expression domain *D*; and $A_s^r$ is the chromatin accessibility score for region *r* for the *s*th developmental stage. For expression domains with reasonably performing trained models, we were able to refine our crude, preliminary assignment of putative enhancers to that domain to those with model support.

## RESULTS

### Motifs and DNA accessibility together accurately predict genome-wide TF–DNA binding

**Figure 1.**
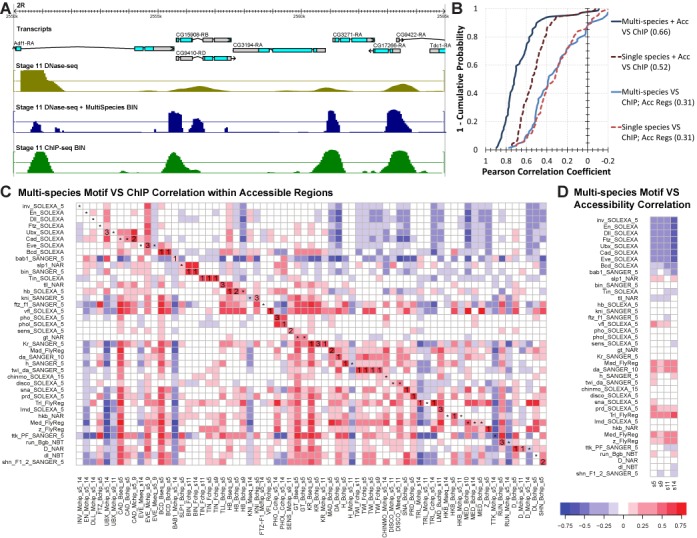
Motifs and DNA accessibility together accurately predict TF–DNA binding. (**A**) Scoring profiles around a typical *Drosophila* gene locus. The positions of the genes (light blue) are shown in this 15 kb browser view. The scoring profiles depicted are, from top to bottom, the chromatin accessibility from DNaseI-seq of the stage 11 embryo, the multi-species motif scores of BIN within accessible regions (top 10% of accessible windows), and finally the DNA binding of BIN from a ChIP-seq experiment in the stage 11 embryo. (**B**) Inverse cumulative frequency distributions for four evaluations. Each line plots for a given correlation value (x-axis), the percentage of the 69 ChIP sets (y-axis) that are greater than that correlation value. The evaluations using multi-species (single-species) scores are solid blue (dotted red) lines. The darker lines represent evaluations between ChIP scores and ‘motif + accessibility’ scores, while the lighter lines represent evaluations comparing ChIP scores to ‘motif only’ scores in only accessible regions. (**C**) Pairwise correlation between ChIP scores and motif scores within accessible genomic regions. The columns of the heatmap represent the 69 ChIP named for the assayed TF, laboratory source, and developmental stage. The rows represent the experimentally determined motifs of the 40 corresponding TFs. Each cell is colored for the Pearson correlation between 2000 windows selected to have 1000 non-coding, accessible ChIP profile peaks and 1000 non-coding, accessible random regions. In a cell where the motif and ChIP profile represent the same TF, the rank (or star if rank>3) of that motif by correlation among the 40 TFs is enumerated. (**D**) Correlation of accessibility scores with motif only scores from different motifs. Similar to (C) except instead of using scores of ChIP profiles we used the four DNaseI-seq chromatin accessibility profiles named for their developmental stage. The Spearman correlation is calculated on 2000 windows selected to have 1000 non-coding accessibility peaks and 1000 non-coding random regions.

We first examined the pairwise correlation among 69 ChIP data sets from early development covering 40 TFs (SM2). Consistent with previous studies (35,42), we observed that most pairs of TFs have highly correlated binding profiles (Supplementary Figure S1). This is commonly attributed to the strong influence of chromatin accessibility on TF binding (18) and supported by Supplementary Figure S2. Given this observation, our ‘motif + accessibility’ scoring method only keeps the computationally predicted motif scores (SM1) that fall within the most highly (above 90th percentile) accessible genomic segments (SM3). It combines the static sequence-encoded information about TF-binding potential with the dynamic, tissue or stage-specific data from chromatin accessibility. Figure 1A shows an example where four major ChIP peaks of the TF *Biniou* (BIN) in a genomic locus are clearly predicted by this method.

Our ‘motif + accessibility’ scoring method was evaluated as previously described (12), using the strongest 1000 ChIP peaks and 1000 randomly selected non-coding segments (SM4). We noted a very high level of agreement between our predicted scores and ChIP profiles—the average Pearson correlation coefficient (PCC) over the 69 data sets was 0.66 (Figure 1B), with 61 of the 69 data sets exhibiting PCC > 0.5 (*P*-value of such a PCC on 2000 data points is <1E−127). We showed that incorporating multi-species information into our scores provides an advantage over single-species in predicting TF binding profiles (Figure 1B), an intriguing observation since the ChIP data reflects binding specific to *D. melanogaster* (see ‘Discussion’ section). Importantly, our predictions were highly informative of TF binding levels, even when we restricted our evaluations to only accessible regions (Figure 1B and Supplementary Note 1). Figure 1C and Supplementary Figure S3 show that in most cases the score predictions from the corresponding motif exhibits greater concordance with its ChIP data set than the predictions from motifs of different TFs. Our results support the premise that TF motifs together with accessibility data can be used to approximate TF–DNA binding profiles in instances where ChIP assays on multiple TFs may be impractical.

### Several motifs are strongly correlated or anti-correlated with accessibility

We hypothesized that some of our predicted motif scoring profiles of TFs might significantly correlate with accessibility, as might be anticipated for pioneer factors that establish a permissive chromatin state (43). In fact, several motifs showed strong positive correlation (Figure 1D); including known pioneer factors such as *Trithorax-like* (TRL)(44) and *Vielfaltig* (VFL)(45), also called *Zelda*, as well as basic helix–loop–helix TFs such as *Medea* (MED), and *Mothers against dpp* (MAD) (SPCC ≥ 0.25 over 2000 windows, *P*-value ≤ 1E−31). Surprisingly, many of these correlations are comparable to or even better than the correlations between the motif based scores and their corresponding ChIP profiles (Supplementary Table S4). We observed clear trends in time-dependent roles of motifs in predicting accessibility, e.g. VFL is correlated primarily at the earliest stages of development and TRL increases in importance during later stages (Figure 1D), as has also been reported previously (12,45). Interestingly, there were also several homeodomain TFs, including *Bicoid* (BCD), *Caudal* (CAD), *Engrailed* (EN) and *Invected* (INV) that are anti-correlated (SPCC ≤ −0.35 over 2000 windows, *P*-value ≤ 1E−56) with chromatin accessibility, a phenomenon for which we are unaware of any suggested mechanisms in the literature. Overall, our analysis of accessibility data strongly suggests the potential of a motif-based computational method to approximate accessibility profiles, as long as the relevant motifs can be identified for the cell type of interest.

### Identification of TFs regulating spatio-temporal expression domains

**Figure 2.**
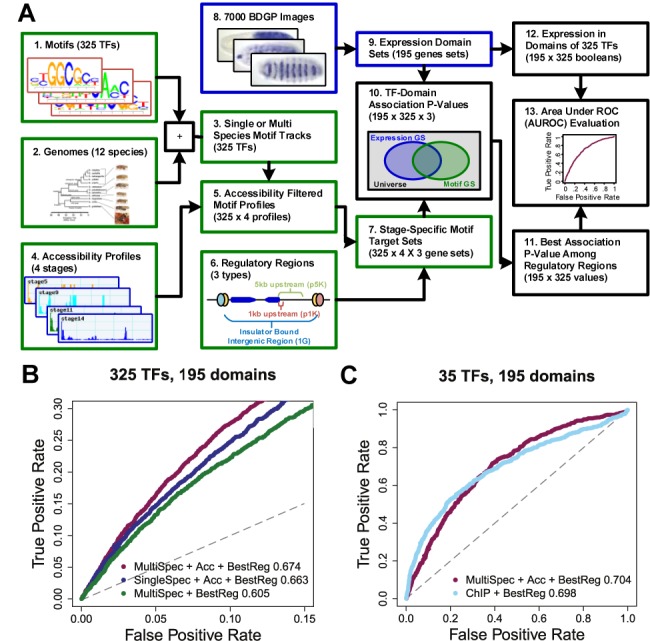
Identifying TF regulators of developmental expression domains. (**A**) Method for discovery of TF–domain associations. The association tests are performed between 195 gene sets defined by BDGP expression annotations and gene sets formed from motif scans of 325 transcription factor motifs filtered by chromatin accessibility from four developmental stages with three different regulatory region definitions. The best regulatory region definition is chosen and the associations are evaluated by the expression of the transcription factors in the expression domains. Additional details of the procedure are found in the text and Supplementary Methods (SM5–SM8). (**B**) Comparison of association methods by area under receiver operator curves (AUROCs). The best method ‘MultiSpec + Acc + BestReg’ of calculating TF–domain associations uses multi-species motif scans instead of single species, an accessibility filter instead of none, and merges best results across three different regulatory region definitions (‘p5K’, ‘p1K’, ‘IG’). The three ROC curves are calculated using domain specific expression of the TF as the ground truth and the region of low false positive rate is plotted. The AUROC is reported in the legend and is 0.674 for our best method. The gray dotted line shows the expected ROC. (**C**) Comparison of best association method using multi-species scores filtered by accessibility to equivalent ChIP-based method. This analysis is the same as above, but is restricted to 35 TF/motifs for which we have ChIP data.

A hallmark of modern regulatory genomics is the ability to use TF binding profiles to investigate a gene's *cis-*regulatory logic (46), and conversely, to determine biological processes (e.g. tissue types in various developmental stages) that are controlled by a TF (e.g. (47)). We consider the latter application here, asking if motif score profiles can be used, as ChIP profiles might be, to assign functional roles to individual TFs. To this end, we created a pipeline for quantifying TF–domain associations (Figure 2A). We extracted gene sets from 195 distinct expression domains as defined by the gene expression atlas of *Drosophila* embryonic development (SM5). We scored a TF–domain association by the most significant overlap between the expression domain gene set and the three different TF target sets created from the TF's multi-species motif profile, the stage specific chromatin accessibility, and one of our possible gene regulatory region definitions (p1k, p5k, IG) (see SM6 and SM7).

To evaluate our TF–domain association pipeline, we collected 3412 (TF, domain) pairs to use as a proxy for the ground truth where the TF gene is specifically expressed in the domain. We then evaluated our pipeline by comparing its (TF, domain) pair predictions to the ground truth and reporting the area under receiver operator curve (AUROC). We found that our pipeline using ‘multi-species motif + accessibility’ scores (AUROC = 0.67), was (a) slightly better than when using motif scores from *D. melanogaster* only (AUROC = 0.66), and (b) significantly better than when ignoring accessibility information (AUROC = 0.605) (Figure 2B). Additionally, the strategy of opportunistically taking the best of three regulatory region definitions (p1K, p5K, IG) was found to be slightly superior to considering any one definition alone (Supplementary Figure S5).

At a *P*-value threshold of 1E−7 (Bonferroni corrected *P*-value < 0.0064), 5716 (TF, expression domain) pairs were designated as significantly associated, with a true positive rate of 24% and a false positive rate of 8% based on the TF presence in that domain. The low false positive rate indicates that if a TF–domain association is not supported by the TF's expression, our method mostly does not predict that association. Overall, our method predicted that 24% of the time, a TF expressed in a domain plays a significant role in regulating that domain. This is expected since we sought primarily to identify TFs with broad regulatory roles spanning several target genes of the domain.

We then asked how the predicted associations compare to similar associations that are inferred if we use ChIP data in place of motif scores. We analyzed ChIP data sets from early embryonic development that span 35 distinct TFs (Supplementary Table S1), and predicted TF–domain associations among all possible 35 × 195 = 6825 pairs, using the same approach as for motif scores. Using TF expression annotations as ground truth, we were surprised to find that the AUROC of ChIP-based predictions (0.698) was comparable to the motif-based method (AUROC = 0.704, Figure 2C), all other aspects of the evaluation being the same. We noted the ChIP-based method to have increased sensitivity at high levels of specificity, while the motif-based method recovered more true TF–domain relationships at a 50% false positive rate. The TF–domain associations predicted by these two approaches overlap significantly, with 53% of the 567 ChIP-based associations being recovered from 710 motif-based associations (*P*-value < 1E−162) (Supplementary Figure S6). This analysis suggests that motif-based approximations of TF–DNA binding profiles are not only strongly similar to ChIP-based profiles, but also that they may be as useful as ChIP data for assigning TFs their regulatory roles in specific expression domains.

We next focused our attention on those significant TF–domain associations (identified above) that were supported by TF expression data. We considered the expression support of the TF in the specific domain, in related domains defined from the anatomical term hierarchy from FlyBase (48), and by ubiquitous TF expression in the particular developmental stage (SM8 and Supplementary Note 3). This compendium included 1232 TF–domain associations (Table 1), with about 14 candidate regulators per domain (Supplementary Table S8), while each TF is assigned to about six expression domains (Supplementary Table S9). Table 2 shows a selection of TFs with the most domain associations per developmental stage within this compendium. These include known pioneer factors VFL and TRL. Our method also identifies *Zeste* (Z) and *Adh transcription factor 1* (ADF1) as important regulators of many expression domains in multiple developmental stages; both TFs have been linked to regulating polycomb group complexes by binding to polycomb response elements throughout the genome (49,50). Many of our TF–domain associations, such as *Brinker* (BRK) regulating embryonic ventral epidermis, *Twin of eyeless* (TOY) regulating embryonic brain, and *Serpent* (SRP) regulating embryonic/larval fat body, are supported through phenotypic data of mutant alleles curated by FlyBase (Supplementary Table S10).

**Table 1.**
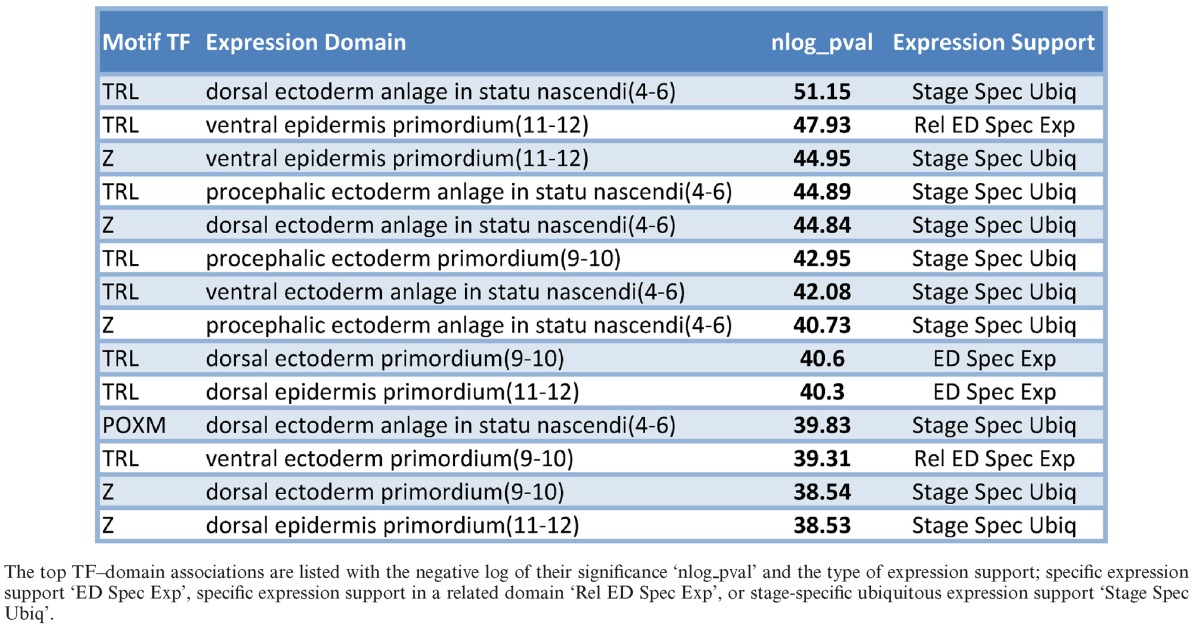
Most significant TF–domain associations

**Table 2.**
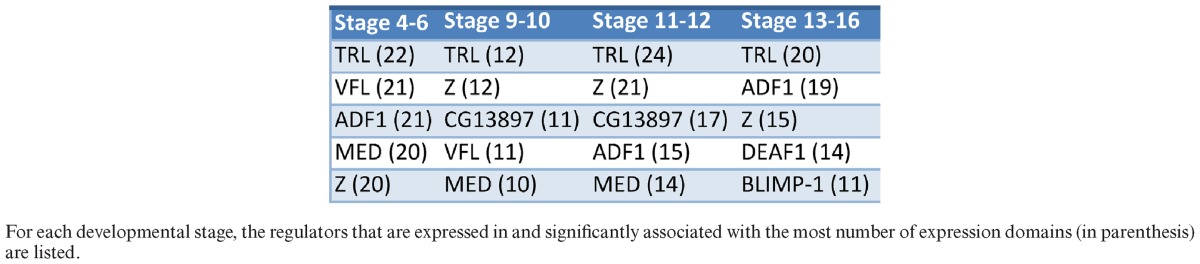
Table of shared regulators

**Figure 3.**
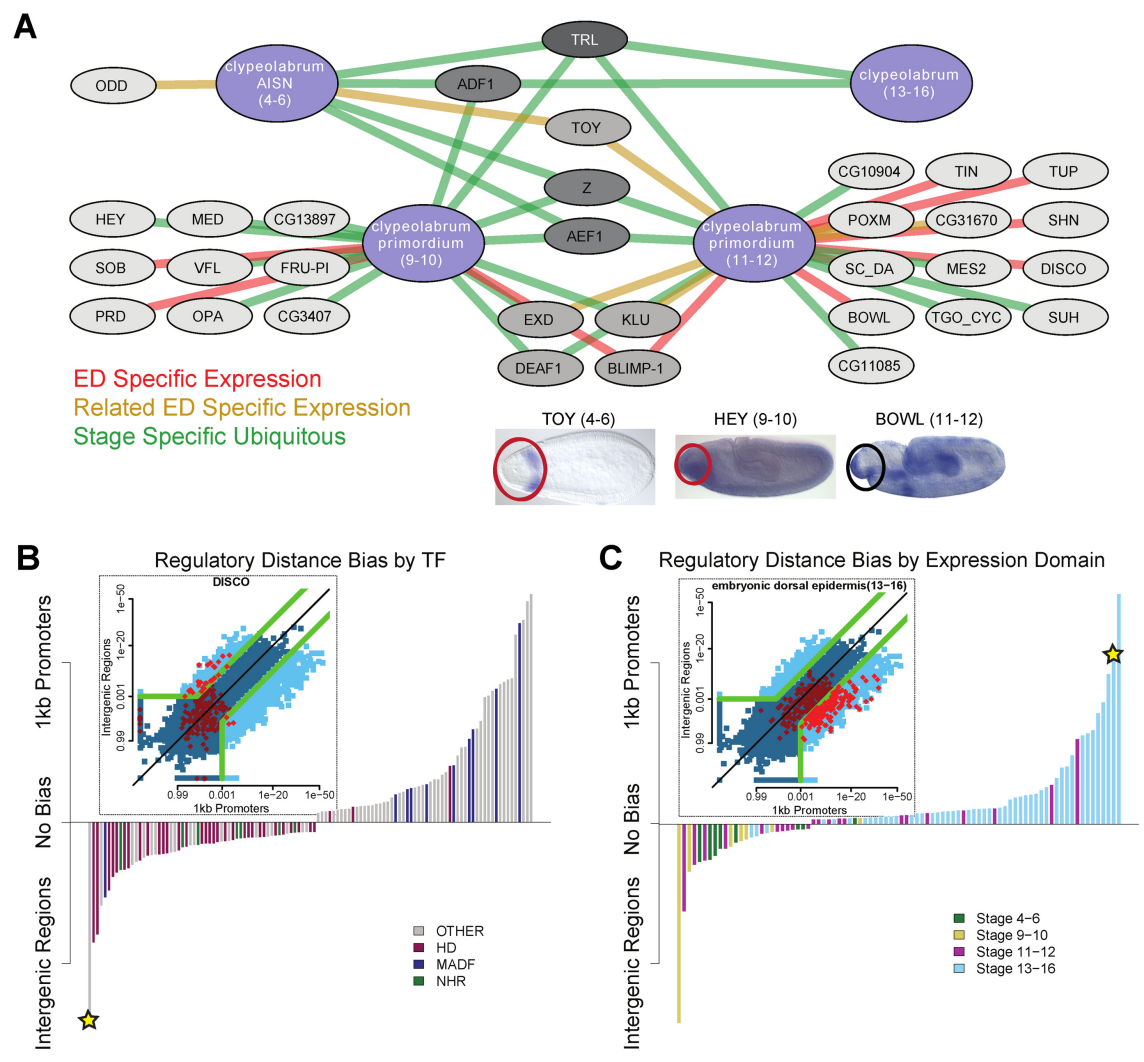
Applications of TF–domain association discovery. (**A**) Clypeolabrum network (66) example. Four expression gene sets from BDGP related to clypeolabrum development in the early embryo are shown as blue nodes ordered counter clockwise from the top left. Grey nodes indicate TFs. Edges are drawn when the corresponding TF–domain association is significant (<1E−7). TF nodes are colored from light to dark by the number of association edges they have. Edges are colored by the type of expression support indicated in the legend and have been filtered to remove TFs with similar motifs (SM9). TFs are clustered by the set of clypeolabrum expression domains they regulate. Below the network are *in situ* images of three different TFs at different stages whose clypeolabrum associations are supported with consistent expression. The clypeolabrum (black circle) emerges from the procephalon (red circles). (**B**) Distribution across binding domains families for TFs with greatest regulatory region biases. Each bar represents a different TF colored by its DBD family and height indicting the statistical strength of the bias between the proximal ‘p1K’ regulatory region and the more distal, insulator defined ‘IG’ regulatory region. The starred transcription factor is shown in detail in the inset plot with the *P*-values of the two methods for all TF–domain pairs in blue and for the 195 DISCO–domain pairs in red. Only points outside of the green lines are considered to be significantly biased. (**C**) Distribution across stages for expression domains with the greatest regulatory bias. Same as (B) with inset plot showing 325 TF associations with embryonic dorsal epidermis (stage 13–16).

The above compendium of TF–domain associations is made available through an easy-to-navigate online interface at [http://veda.cs.uiuc.edu/B1H_GRN] and described in Supplementary Note 4. Navigation of the compendium allows us to describe the development of a tissue type over time in terms of the main TFs involved in the regulatory process. Figure 3A and Supplementary Figure S9 illustrate this with the predicted regulatory network of clypeolabrum (larval feeding organ) development through the stages ‘*anlage in statu nascendi* (stages 4–6)’, ‘primordium (stages 9–10)’, ‘primordium (stages 11–12)’ and the mature clypeolabrum (stages 13–16). The networks show transcription factors that are predicted to be related to all developmental stages (TRL, ADF1), primarily early stages (e.g. *Adult enhancer factor 1* (AEF1), *Sister of odd and bowl* (SOB), VFL) or only later stages (*Tinman* (TIN)), based on motif analysis as well as expression data.

We next show that motif-based TF–domain associations can provide systems-level insights into *cis-*regulatory architecture. Recall that the TF–domain associations are based on motif scans involving three different definitions of regulatory regions—1 kb upstream (‘p1K’), 5 kb upstream (‘p5K’) and intergenic with insulator site boundaries (‘IG’)—and opportunistically using the definition that gives the strongest association for each TF–domain pair. We noted that ∼56% of all significant associations were derived from the p1K definition, while for ∼28% of associations the strongest signal came from the ‘IG’ definition (Supplementary Table S11), suggesting that the compendium is not dominated by promoter signals or distal signals only.

We asked if certain TFs tend to have stronger regulatory signals in one of these classes of regulatory regions versus others (51,52). If a TF–domain association was significant when examining one class of regulatory regions and not significant in a different class, we deemed the association to be specific to the former class (SM10). Each of the TFs, TRL (Supplementary Figure S10), *Zeste* (Z), ADF1, *Deformed epidermal autoregulatory factor-1* (DEAF1), CG4360, *Klumpfuss* (KLU), MAD and MED, were found to have p1K-specific associations, i.e. associations seen only in promoter scans, with over 50 expression domains but no IG-specific associations, i.e. associations seen only in broader scans around the gene (Supplementary Table S12). *Zeste* has been demonstrated to frequently bind proximally to a gene and facilitate communication with distal enhancers (53). Additionally, *Disconnected* (DISCO), *Extradenticle* (EXD), *Goosecoid* (GSC) and BCD showed IG-specific associations with tens of expression domains (Figure 3B), but few or no p1K-specific associations, thus pointing to dominance of distal regulatory signals for these TFs. Overall, we found that as a class, homeodomain TFs have a preference for acting via distal regulatory regions, consistent with (51). We also found several predominantly late-stage expression domains that prefer TF associations with proximal regulatory signals (Figure 3C, Supplementary Table S13) and several early stage domains that are skewed toward distal signals, pointing to an architectural difference between early and later developmental regulation that had not been previously appreciated.

### Enhancers associated with expression domains

High throughput chromatin state (e.g. DNaseI hypersensitivity) data has been used to identify putative enhancers in the genome (4,6,10,27,29,54,55). However, these approaches typically do not associate enhancers with genes and expression domains. We sought to predict the target gene and expression domain of putative enhancers using enhancer activity models that incorporate the predicted TF motif profiles and TF–domain associations from above.

**Figure 4.**
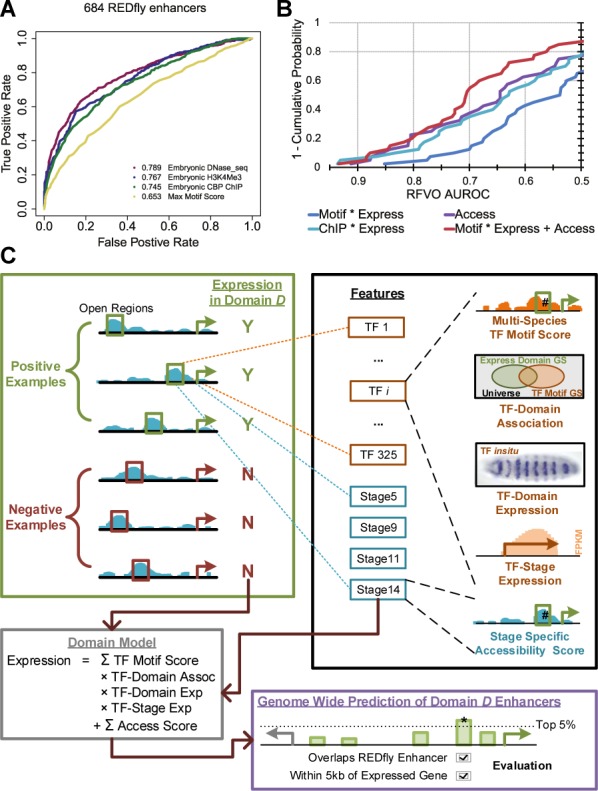
Modeling expression domain enhancers. (**A**) ROCs for methods of detecting 684 REDfly enhancers from 684 negative sequences. Using the chromatin accessibility score from embryonic DNaseI-seq data (AUROC 0.789) is the best method. It is more discriminative than using scores for the presence of chromatin mark H3K4Me3, the binding of transcriptional co-activator CBP, or the maximum of 325 multi-species motif scores. (**B**) Comparisons of four different models. For each type of model, we calculated the AUROC using the RFVO test set on each of the 40 expression domains (SM13). The distribution of these forty values is visualized with the x-axis showing a particular value of the AUROC and the y-axis indicating the percentage of the domains with a stronger AUROC. Of the four models compared, the best model, ‘Motif * Express + Access’, combines 325 motif based features with four accessibility based features in a linear model (see panel **C**). The ‘Motif * Express’ and ‘Access’ models use a subset of features from the best model, and the ‘ChIP * Express’ model (SM14) uses one feature from each of the 69 downloaded ChIP data sets. (**C**) Training domain specific models of enhancer expression. Our linear model combines each putative enhancer's accessibility features with TF features that are the product of the motif score, the importance from our compendium of the TF in regulating the domain, and the TF's expression from *in situ* annotations and from RNA-seq data. ‘Good’ models (RFVO AUROC > 0.7 or Test AUROC > 0.6) are applied to every accessible window in the genome. The top 5% of windows that predict the given domain expression that are within the regulatory region of a gene expressed in that domain are predicted as domain-specific enhancers. We evaluate these predictions by their agreement with REDfly enhancers.

We evaluated several types of genome-wide assays to identify the best method for locating putative enhancers, using 684 non-overlapping REDfly enhancers (56) as a benchmark (SM11). Open chromatin, as indicated by high accessibility scores, was found to be the best method with an AUROC of 0.789 (Figure 4A). Occupancy profile of the general transcriptional co-activator *CREB Binding Protein* (CBP), as well as histone marks associated with enhancer and promoter regions (H3K4Me3, H3K4Me1, H3K9Ac, H3K27Ac) were also predictive (Supplementary Figure S11), while phastCons scores of evolutionary conservation (57) and methods based on combining motif scores (58) were considerably worse at discriminating REDfly enhancers. These observations motivated our decision to define our set of putative enhancers as those non-overlapping 500 bp segments that are among the top 10% most accessible regions in any of the four developmental stages 5, 9, 11 and 14. (See SM12 for additional criteria used to further restrict this set.) We henceforth refer to this set of accessible segments as ‘open regions’.

As a preliminary assignment of regulatory activity, we annotated each open region as a potential enhancer of its two nearest neighboring genes and all of their expression domains, discarding open regions for which neither neighboring gene is annotated with one of the 195 expression domains of interest. This defined a set of ∼24 000 open regions as candidate enhancers, each with one or two genes defined as its potential targets and one or more expression domains corresponding to those genes as its potential domains of activity. On average, about 14 expression domains were tentatively assigned to each enhancer.

To further refine these tentative domain assignments, we learned computational models (classifiers) capable of predicting expression driven by an enhancer. This requires training sets of ‘positive’ and ‘negative’ examples, i.e. open regions known to drive or not drive expression in a particular domain. Reliable training sets of this type are rare for most expression domains. Enhancers from the REDfly database may be used for training models, but this would limit the model training to relatively few expression domains. Instead, we chose to train models on the numerous open regions putatively assigned to each domain, so that the positive (negative) training sets are likely to be enriched in (depleted of) enhancers of an expression domain (SM13, Supplementary Figure S14). Use of these ‘noisy training sets’ also allowed us later to treat REDfly enhancers as ‘unseen’ test data for evaluating the models.

For each expression domain, we trained a ‘complete’ linear model to discriminate positive and negative open region examples using features that correspond to each of the 325 TFs in our collection and each of the four stages of development (see ‘Materials and Methods’ section). Each TF-related feature was the product of four quantities: the multi-species motif score of the TF in the open region, the strength of statistical association between the TF's motif and the expression domain, the expression annotation of the TF's gene in the given expression domain, and the RNA-seq expression level of the TF's gene in the appropriate developmental stage. Accessibility scores of the open region in each of the four developmental stages were also included as features describing the open region (Figure 4C).

While a separate model was trained for each of the 195 expression domains, we first focused on evaluating our approach on 40 domains for which there were at least 10 REDfly enhancers annotated with that expression domain, affording us a reasonable test of the model on unseen data. For these domains, we created ‘REDfly versus Open Regions’ (RFVO) test sets comprising open regions that overlap REDfly enhancers annotated with the domain and those that do not overlap any REDfly enhancers nor have either neighboring gene annotated with the domain. (A more stringent test set is described in Supplementary Note 5.)

Our ‘complete’ linear models using the above-mentioned features exhibited an AUROC of at least 0.7 on RFVO test sets from 21 of the 40 expression domains. For the remaining 155 expression domains, REDfly evaluations were not possible and AUROCs were obtained using ‘left-out’ test sets from the noisy training sets described above. Fifty-six of these expression domains exhibited a test AUROC of at least 0.6, a level of discrimination observed on only 3 of 155 domains in negative controls (Supplementary Note 6). Thus, we learned accurate models for 77 of the 195 expression domains overall (Supplementary Table S15). We used the same evaluation framework to compare the ‘complete’ model to simpler variants that ignored certain types of features. For instance, we found the complete model to accurately predict more expression domains than analogous linear models that use only motif features or only accessibility features (Figure 4B). The advantage of using motif features was most conspicuous for expression domains prior to developmental stage 13 (Supplementary Figure S15). We also compared the linear classification method to other classification schemes such as logistic regression and support vector machines, and found it to be marginally better (Supplementary Figure S16).

**Figure 5.**
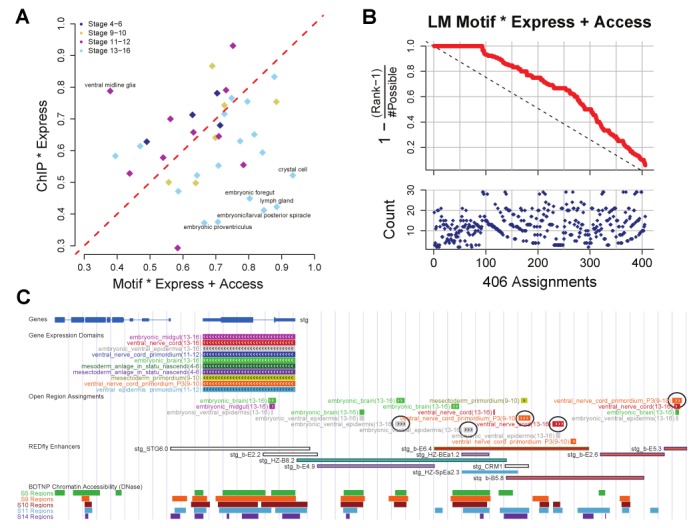
Enhancers model comparison to ChIP and genome-wide predictions. (**A**) Comparison of the RFVO AUROCs. One point is plotted for each of the 40 expression domains with the color indicating its developmental stage. The x-axis (y-axis) is the AUROC of the ‘Motif * Express + Access’ (‘ChIP * Express’) model. Off diagonal points (labeled) are expression domains that find better models using one set of features instead of the other. Motif and accessibility features show greatest advantage over ChIP-based ones for stage 13–16 expression domains. (**B**) Evaluation of 406 open regions that overlap REDfly enhancers. Each open region (x-axis) is near genes annotated with a number of possible expression domains (lower plot, blue dots). We order the possible expression domains by the predictions of our ‘Motif * Express + Access’ models and identify the rank of the ‘true’ expression domain annotated for the enhancer in REDfly. We plot a statistic (upper plot, red line) that achieves a maximum possible value of 1 when the REDfly domain is the best of all possible expression domains of that open region. (**C**) Genome browser view of enhancer predictions near stg gene. The position and structure of genes is shown at the top. At the bottom, the chromatin accessibility from DNaseI-seq of four developmental time points is shown as colored profiles. Each possible expression domain of stg is shown (‘Gene Expression Domains’) and color-coded. The ‘REDfly enhancers’ are shown with the fill and border color matching their annotated gene expression domains. Finally, the ‘Open Region Assignments’ show which expression domains are likely driven by each 500 bp open region. The color and size of the open region box indicate the driven expression domain and the significance of the prediction. Five different open regions are circled where the most significant expression domain prediction is consistent with the annotation of an overlapping REDfly enhancer.

Since our approach uses computationally predicted TF–DNA binding, it is reasonable to compare it to a baseline that utilizes TF–DNA binding data from ChIP experiments in a similar manner. To this end, we trained an alternative classifier where TF-related features utilized 69 publicly available genome-wide ChIP profiles rather than the 325 motif profiles computed by us (SM14). Surprisingly, motif-based models performed accurately on more expression domains than the ChIP-based models (Figure 4B, Supplementary Figure S17), suggesting that the use of computationally characterized TF–DNA binding features spanning more TFs is better than relying on experimentally characterized occupancy for fewer TFs. On closer examination, we noted that an improved performance of motif-based models over ChIP-based models frequently corresponded to expression domains from developmental stages 13–16 (Figure 5A). This may be because of poor temporal resolution of these stages in the available ChIP data or because the crucial TFs of these later stages have not yet been subjected to ChIP assays.

We next attempted to assign expression activity to putative enhancers using the motif-based models trained as above, focusing on the 77 expression domains for which such models were assessed to be accurate. We attributed an expression domain to an open region if one of the neighboring genes is annotated with the domain and the complete model for the domain scored the open region in the top 5% of all 23 529 open regions genome-wide. This resulted in a compendium of 7824 high-confidence enhancer activity predictions spanning 4197 open regions. Over 30% (2354) of these predictions involved putative enhancers located >5 kb away from the target gene.

A large number of activity predictions corresponded to annotated REDfly enhancers, even though these enhancers had not been used in training models. We used these REDfly enhancers to further evaluate the accuracy of genome-wide enhancer activity prediction. For each REDfly enhancer, we examined the strength of its association with each possible expression domain (as predicted by the appropriate model) and found that the experimentally annotated expression domain ranked first significantly more often than expected by chance (Figure 5B). This result was stronger with predictions by the motif-based models than with equivalent predictions by ChIP-based models (Supplementary Figure S18). One successful example of our enhancer activity assignment procedure comes from the *string* (stg) gene locus (Figure 5C). In this region, there are a number of REDfly enhancers annotated to drive expression in the ventral nerve cord and the ventral epidermis. We highlight five open regions in this locus whose predictions for domain specific expression agree with the known expression patterns of overlapping REDfly enhancers.

## DISCUSSION

We have demonstrated here the utility of a comprehensive collection of TF motifs in annotating the regulatory genome of an organism at multiple levels of resolution: binding loci of a TF, identities of major regulators of expression domains, and enhancer activities. At each level, we assessed our methodology with independent experimental evidences. We validated our computationally predicted binding profiles by direct comparison to ChIP within a locus (Figure 1A) and by having them separate ChIP peaks from non-peaks (Figure 1B and C). We demonstrated our ability to identify TF regulators of an expression domain by comparing to manual annotations of experimentally determined TF expression (Figure 2B), as well as relying on ontology-based spatial-temporal relationships among domains (Supplementary Figure S7). Finally, we evaluated our ability to predict the domain specific activity of putative enhancers with annotations of known enhancer activity from *in situ* hybridization experiments curated by the REDfly database (Figure 5B and C).

In light of available data in *D. melanogaster*, we assessed multiple methodological choices and adopted the best available strategy for each level of *cis-*regulatory annotation. Importantly, we noted that the estimated accuracy of these annotations using our motif-based approach is comparable to those using ChIP data sets available today. This observation has major practical implications, as it relies on a single accessibility assay per cell type as an alternative to the popular paradigm of characterizing ChIP binding profiles for every TF in the cell type of interest. Our work goes beyond an exploration and demonstration of methodology, to actually create a comprehensive regulatory map, pertaining to dozens of cell types in the developing *D. melanogaster* embryo. This map, made available through an easy-to-use online interface, can be used by biologists studying specific aspects of embryonic development at a transcriptional level. In constructing this map, we also identify several interesting trends and reported systems-level insights into regulation in development.

Our first analysis involved the use of motifs to predict TF binding profiles. Unlike previously reported methods that trained free parameters from ChIP data, (9,12,21), our prediction approach was completely free of hand-tuned parameters. Consistent with our findings in (24) on only six TFs, we noted that evolutionary conservation, measured by a phylogenetically weighted average score of motif presence in orthologous segments, provides substantial improvements in the accuracy of occupancy prediction for dozens of transcription factors. We speculate that this is because evolutionary conservation serves as a proxy for the contextual information that is necessary for *in vivo* TF binding.

We noted above that the paradigm of using motif-based computational predictions will rely upon cell type-specific accessibility profiles, obtained using experimental methods (59–61). Additionally, we noted very strong positive and negative correlations between motif presence and accessibility. The informative motifs were often stage-specific, e.g. VFL correlated strongly in the earliest stage analyzed and poorly in the last stage, consistent with its temporal expression profile. Thus, in principle, future methods may be able to utilize expression data on TFs along with their motif profiles to predict approximate accessibility profiles in a stage-specific manner, which then may be utilized to predict stage-specific occupancy profiles for each TF.

Our second goal was to use motifs to create a large compendium of statistical associations between regulatory TFs and their target tissues and cell type-specific programs. We noted that our motif-based approach has roughly the same accuracy as a ChIP-based approach, again arguing for the proposed alternative paradigm at the heart of this work. The compendium, with its separate predictions for the same tissue in different stages of its development, also allowed us to observe temporally changing gene regulatory networks, such as the one for clypeolabrum development (Figure 3A) through the four stages. We found that the best strategy for predicting associations was to examine all three classes of control regions (1 kb upstream, 5 kb upstream, and broad intergenic territory) rather than to limit ourselves a priori to one of these classes as is typically done in the literature. This also enabled us to identify biases exhibited by certain TFs and expression domains for either proximal or distal *cis*-regulation on its target genes. We noted that TFs with widespread regulatory functions were the ones with a proximal bias, while biases for the broader control region tended to be exhibited by Homeodomain TFs. Several late-stage domains were found with a bias for proximal control regions in the compendium (Supplementary Table S13), pointing to the possibility that later stages of development are less dependent on distal enhancers, compared to earlier stages.

With high throughput technologies becoming the norm (46) for predicting enhancer locations, the challenge of the day has shifted to annotation of enhancer function. As one of the most ambitious attempts to date at tackling this challenge, a major contribution of our work is the construction of models for assigning activity to enhancers for as many as 77 of the 195 expression domains. Prior work in the field has attempted this with one (24–26) or a handful (2,3,27,28) of domains. These earlier models are constructed from suitable training sets of validated enhancers experimentally associated with that expression domain (2,6). As such training data sets are generally not available for most tissues, we considered the possibility of defining ‘noisy’ training sets of enhancers active in an expression domain based on their accessibility and the distance and expression of their nearby gene. This pragmatic choice allowed us to successfully build regulatory maps for many domains beyond the handful with validated enhancers.

We found our motif-based approach to annotate enhancer activity to be as effective as an analogous approach based on ChIP data. This is not a fair comparison since one method uses motifs for 325 TFs and the other relies on ChIP data for 69 TFs. However, the comparison should be interpreted in light of the costs of generating equivalent data for the two methods, a single accessibility profile for the domain versus hundreds of ChIP-seq experiments.

Our work demonstrates that understanding of cell type specific regulatory networks and elements can be obtained from combining only gene expression and chromatin accessibility data with computationally predicted profiles of TF binding. We have previously applied a motif-based approach to identify major TFs involved in transcriptional programs to systems outside of *Drosophila* development where chromatin structure data was not available, including other insect genomes (16,62), and vertebrate genomes such as mouse (63), zebra finch (64) and stickleback fish (65). The results in this paper suggest that with the increasing availability of accessibility data, the efficacy of a motif-based approach is expected to improve, especially for vertebrate genomes where such data can greatly reduce the search space for *cis-*regulatory signals. The methods presented here, which attempt to reconstruct the maximal regulatory network from minimal experimental requirements will be particularly useful to biologists who study non-model organisms or specific cell types that are not investigated by well-funded projects such as ENCODE.

## SUPPLEMENTARY DATA

Supplementary Data are available at NAR Online.

SUPPLEMENTARY DATA
