# Supplementary material for: Integrating motif, DNA accessibility and gene expression data to build regulatory maps in an organism
Source: Nucleic Acids Res. 2015 Mar 19;43(8):3998–4012. doi: 10.1093/nar/gkv195 (PMC4417154; doi:10.1093/nar/gkv195)
Supplement: SUPPLEMENTARY DATA [file supp_43_8_3998__index.html]

Integrating motif, DNA accessibility and gene expression data to build regulatory maps in an organism — SUPPLEMENTARY DATA 

# Integrating motif, DNA accessibility and gene expression data to build regulatory maps in an organism

## SUPPLEMENTARY DATA

**Files in this Data Supplement:**

- SUPPLEMENTARY DATA
- SUPPLEMENTARY DATA
