# Supplementary material for: Integrating motif, DNA accessibility and gene expression data to build regulatory maps in an organism
Source: Nucleic Acids Res. 2015 Mar 19;43(8):3998–4012. doi: 10.1093/nar/gkv195 (PMC4417154; doi:10.1093/nar/gkv195)
Supplement: SUPPLEMENTARY DATA [file supp_gkv195_nar-02821-n-2014-File009.docx]

**SUPPLEMENTARY METHODS**

**Supp Methods SM1. Creating Motif Scores for Genomic Windows**. Motifs for 325 transcription factors are available from FlyFactorSurvey (1) and listed in Table S2. Motifs scans were performed on the 12 *Drosophila* genomes after masking each genome with Tandem Repeat Finder (2). The Stubb_fixed program was run for each motif and each genome with a motif transition probability of 0.0025, as in (3), producing motifs scores for 500 bp-long genomic windows with 50bp shifts. The 5kb upstream regions of 86 developmental genes in *Drosophila* were used to train the background model. Multi-species motif scores were produced by converting the motif scores in each species to species-specific z-scores, setting negative z-score to 0, and computing phylogenetically weighted averages of scores from orthologous windows across the 12 species using Brownian Motion averaging (3).

**Supp Methods SM2. Creating ChIP Scores for Genomic Windows.** We downloaded 69 ChIP data sets from early embryonic development covering 36 TFs (Table S1). There are five ChIP-seq data sets and 25 ChIP-chip data sets from BDTNP (4), eight ChIP-chip data sets from the Furlong lab (5), and 21 ChIP-chip and five ChIP-seq data sets from the ModENCODE project (6), and 5 ChIP-chip data sets from the Cavalli, Busser, and Rushlow labs (7-9) The raw ChIP data was converted into averaged values for each of our 500bp genomic windows by averaging the maximum read scores from each 50bp subsegment of the window.

**Supp Methods SM3. Creating Accessibility Scores for Genomic Windows.** DNaseI-seq chromatin accessibility data was downloaded from BDTNP (10) from five stages of embryonic development (5, 9, 10, 11, and 14). These tracks were converted from release 4 to release 5 using the liftOver [http://hgdownload.cse.ucsc.edu/downloads.html] tool from UCSC Genome Browser. Using the same procedure as the ChIP data, we converted each of these raw “read scores” into averaged values for each of our 500bp genomic windows. The top 10% of windows within each developmental stage (11) are considered accessible in all analyses.

**Supp Methods SM4. Calculating Correlations between Two Scores.** Each correlation was calculated between a primary score (e.g. ChIP score) and a secondary score (e.g. motif score). We select 2000 non-overlapping windows from the non-coding genome, 1000 windows with the highest primary scores as “peaks” and 1000 random regions as “non – peaks”. If the windows are restricted to accessible regions, 2000 windows are chosen in the same way with the additional criteria that they also be in the top 10% of chromatin accessibility for the earliest developmental stage that overlaps the interval of the ChIP profile. The secondary scores for the 2000 windows are also extracted. If the secondary score is a motif score filtered by accessibility, the secondary scores of windows not within the top 10% of accessibility for the earliest overlapping stage are set to 0. The Pearson correlation is calculated between the two scores over the 2000 windows.

**Supp Methods SM5. Creating Gene Expression Sets.** Two files were downloaded from BDGP (12) [<http://insitu.fruitfly.org/insitu-mysql-dump/insitu.sql.gz> and <http://insitu.fruitfly.org/insitu-mysql-dump/insitu_annot.csv.gz>] and each gene ID, developmental stage ID, and BDGP annotation term triplet was extracted. Expression domain sets were created by merging all gene IDs for any stage-term pair. Expression sets that were too general (>1500 genes), too small (<20 genes), or did not have a spatially descriptive annotation term were removed. An additional expression domain set of 168 genes with anterior-posterior segmentation patterns from (3) was created for stage 4-6 and labeled with the term “ap_genes”.

**Supp Methods SM6. Creating TF Target Sets.** A TF target set is created using the window-based scores of a particular scoring method (multi-species motif, single species motif, or ChIP) that may be set to 0 in inaccessible windows if filtered by the chromatin accessibility of some developmental stage. For each gene in the genome, the maximum score of all windows of its regulatory region is recorded. The 400 genes with the best maximum scores are considered the TF target set.

**Supp Methods SM7. Defining Regulatory Regions.** The genomic windows located within 1 Kbp (resp. 5 Kbp) upstream of the transcription start site of a gene for the “p1K” (resp. “p5K”) control region of that gene. To define the “IG” (intergenic) control region, we first downloaded the 1% FDR ChIP-chip data for three insulators (BEAF-32, CP190, and CTCF_C) for early embryonic development (E0-12h) from ModENCODE (6). For each gene, the IG control region includes the gene and extends on either side of the gene by 50 Kbp or until a window in which two of the three insulator proteins are bound, whichever happens first (13). Furthermore, only windows designated as accessible in any of the five developmental stages constitute the IG regulatory region.

**Supp Methods SM8. Related Expression Domains.** We downloaded the anatomical term database from FlyBase (14) [<http://flybase.org/static_pages/downloads/FB2012_06/ontologies/fly_anatomy.obo.zip>] and succeeded in manually mapping 177 of our 195 BDGP annotation terms to a FlyBase anatomical term (Table S5). We created the hierarchical graph of the FlyBase data where the nodes are anatomical terms and the edges represent an annotated relationship between them. Using this graph, we found all of the direct links between any pair of our anatomical terms (or any of their synonyms as defined by FlyBase). We also collected second order relationships between our expression domains where their two terms, T1 and T2, are considered “siblings” if they share the same parent for a particular relationship type (e.g. “develops_from”) and “spouses” if they share the same child node. Finally, we added our own relationship “devs_from” that extracted the core words of the BDGP terms and created relationships between terms with the same core in temporally adjacent developmental stages (e.g. stage 11-12 is adjacent to stage 13-16). This procedure resulted in 1068 pairs of expression domain relatives (Table S7) with 12 different types of relationships (Table S6).

**Supp Methods SM9. Filtering TF-Domain Associations.** For every pair of TFs, similarity between their representative motifs was calculated with MatAlign [Wang and Stormo; <http://stormo.wustl.edu/MatAlign/> ]. Motif pairs with a similarity value less than 1E-4 were defined as “similar”. For any pair of (TF, expression domain) that was found to be significantly associated we asked if information on the TF’s expression supports a functional role for the TF in regulating the expression domain. For this, we defined three levels of support: 1) specific TF expression in the domain, 2) specific TF expression in a related domain, and 3) ubiquitous TF expression in the stage of the domain. The motif association with the domain was quantified by the “MultiSpec + Acc + BestReg” value. For each TF-domain association, we gathered all motifs similar to the TF’s motif and examined their respective TF’s expression in and association with the domain. We discarded the TF-domain association if any similar motif is significantly associated (p-value < 1E-7) and has a higher level of expression support, or if any similar motif is more significantly associated with the domain with the same level of expression support.

**Supp Methods SM10. Regulatory Region Bias.** For every TF-domain pair, we calculated an association p-value separately using the “p1K” control region (1 Kbp upstream) and the “IG” regulatory region, which includes more distal regions. We converted this p-value to the corresponding z-score of the standard Normal distribution. To determine if a TF, *F*, had a bias for regulating via proximal promoters, we first counted the number of expression domain associations out of 195 that were significant (p-value < 0.001) with either definition of control region. We define *N^F^_p1K_* as the number of expression domains associated with *F* where the z-score for the “p1K” definition was at least three greater than the z-score of the “IG” definition. *N^F^_IG_* is the corresponding count where the z-score of the “IG” definition is at least three times greater. We similarly find *N^ALL^_p1K_* and *N^ALL^_IG_* which count the appropriate associations across all transcription factors. These four numbers define the values of a 2x2 contingency table on which we use the Hypergeometric test to quantify the significance of *N^F^_p1K_*. A TF’s bias for regulating its targets via distal sites was tested in an analogous manner. We examined the expression domains for regulatory biases using the same approaches except counting over the 325 motifs.

**Supp Methods SM11. Evaluating Candidate Enhancer Selection Methods.** We selected 684 non-overlapping REDfly enhancers (15) with lengths from 100 to 3000 bp. For each REDfly enhancer, we selected a size-matched, random intergenic region near an early development gene. We then calculated the values of several features for these 1368 regions. We downloaded ChIP-chip data sets for the CREB Binding Protein (CBP) at 11 developmental timepoints and for 6 histone marks for 6 developmental timepoints from ModENCODE (6). We also downloaded the *Drosophila* phastCons track (16) from UCSC Genome Browser [<http://genome.ucsc.edu/> ]. We found the best score in each of the 1368 regions for each feature including phastCons, each of 6 histone marks, CBP, DNaseI chromatin accessibility from BDTNP, and each of 325 multi-species motif scores. Multiple timepoints for CBP, histone marks, and accessibility were combined by selecting the best scoring timepoint per region. The 325 motif scores were summarized by the best per region, the average of all, and the number of significant. These score features are independently evaluated for their ability to identify REDfly enhancers by the AUROC metric.

**Supp Methods SM12. Open Regions Selection.** We selected non-overlapping 500 bp segments with accessibility scores in the top 10% of any of the four embryonic developmental stages. We ignored any segments that overlapped exons or regions of tandem repeats by more than 50% of their length. As an additional filter, we only considered segments whose combined multi-species motif score (sum over all 325 motifs) was above a threshold of 10; this filter was motivated by examining the summed motif score distributions of REDfly enhancers (Figure S12). These “open regions” with neighboring annotated genes where the basis of all modeling and prediction of enhancer function. This set of putative enhancer sequences are highly enriched for gene-proximal locations (≤ 5kb upstream of transcription start sites) (Figure S13, Table S14), similar to (10).

**Supp Methods SM13. Defining Model Training and Test Sets**. For each expression domain, *D*, we created a model training set and testing set. We selected up to 500 open regions that were within 5kb of their neighboring gene annotated with *D*, accessible during the developmental stage of *D*, and did not overlap any REDfly enhancers. We marked ¾ as training positives and ¼ as testing positives. We then selected a matching number of negative examples (open regions whose neighboring genes are not annotated with *D*, accessible during the developmental stage of *D*, and did not overlap any REDfly enhancers) for the training and testing sets. The model is trained and the AUROC of the test set is recorded. If at least 10 open regions overlap REDfly enhancers annotated with domain *D*, additional testing sets “REDfly vs. Open Regions” (RFVO) and “REDfly vs. Enhancers” (RFVE) are created. Both RFVO and RFVE test sets use the open regions overlapping the REDfly enhancers as positives. The negatives for the RFVO test set are sampled from the negatives from the general testing set. For the RFVE set, the negatives are chosen from open regions that are accessible during the developmental stage of *D* and overlap REDfly enhancers that are not annotated *D*. The AUROC for the RFVO and RFVE test sets are also calculated and recorded.

**Supp Methods SM14. Form of the “ChIP*Express” Enhancer Model**. The full enhancer model using ChIP and TF expression data for a domain D can be described as

$$y^{r}=\sum_{c=1}^{69} \alpha_{c}C_{c}^{r} E_{c}^{D} R_{c}^{D}+\beta$$

where $y^{r}$is prediction indicating whether region r is in the positive set, *c* is from the 69 ChIP data sets, and $\alpha_{c}$ and $\beta$ are the domain-specific parameters,. $C_{c}^{r}$ is the averaged ChIP score for the region *r* for the *c*th ChIP set. $E_{c}^{D}$ indicates whether the TF related to the *c*th ChIP dataset is expressed in *D* or in a related expression domain. $R_{c}^{D}$ is the fragments per kilobase of exon per million fragments mapped (FPKM) reported from (17) for the TF related to the *c*th ChIP dataset in the developmental stage related to expression domain D.

**REFERENCES**

1. Zhu, L.J., Christensen, R.G., Kazemian, M., Hull, C.J., Enuameh, M.S., Basciotta, M.D., Brasefield, J.A., Zhu, C., Asriyan, Y., Lapointe, D.S. *et al.* (2011) FlyFactorSurvey: a database of Drosophila transcription factor binding specificities determined using the bacterial one-hybrid system. *Nucleic acids research*, **39**, D111-117.

2. Benson, G. (1999) Tandem repeats finder: a program to analyze DNA sequences. *Nucleic acids research*, **27**, 573-580.

3. Kazemian, M., Blatti, C., Richards, A., McCutchan, M., Wakabayashi-Ito, N., Hammonds, A.S., Celniker, S.E., Kumar, S., Wolfe, S.A., Brodsky, M.H. *et al.* (2010) Quantitative analysis of the Drosophila segmentation regulatory network using pattern generating potentials. *PLoS biology*, **8**, epublish.

4. MacArthur, S., Li, X.Y., Li, J., Brown, J.B., Chu, H.C., Zeng, L., Grondona, B.P., Hechmer, A., Simirenko, L., Keranen, S.V. *et al.* (2009) Developmental roles of 21 Drosophila transcription factors are determined by quantitative differences in binding to an overlapping set of thousands of genomic regions. *Genome biology*, **10**, R80.

5. Zinzen, R.P., Girardot, C., Gagneur, J., Braun, M. and Furlong, E.E. (2009) Combinatorial binding predicts spatio-temporal cis-regulatory activity. *Nature*, **462**, 65-70.

6. Negre, N., Brown, C.D., Ma, L., Bristow, C.A., Miller, S.W., Wagner, U., Kheradpour, P., Eaton, M.L., Loriaux, P., Sealfon, R. *et al.* (2011) A cis-regulatory map of the Drosophila genome. *Nature*, **471**, 527-531.

7. Busser, B.W., Huang, D., Rogacki, K.R., Lane, E.A., Shokri, L., Ni, T., Gamble, C.E., Gisselbrecht, S.S., Zhu, J., Bulyk, M.L. *et al.* (2012) Integrative analysis of the zinc finger transcription factor Lame duck in the Drosophila myogenic gene regulatory network. *Proceedings of the National Academy of Sciences of the United States of America*, **109**, 20768-20773.

8. Nien, C.Y., Liang, H.L., Butcher, S., Sun, Y., Fu, S., Gocha, T., Kirov, N., Manak, J.R. and Rushlow, C. (2011) Temporal coordination of gene networks by Zelda in the early Drosophila embryo. *PLoS genetics*, **7**, e1002339.

9. Schuettengruber, B., Ganapathi, M., Leblanc, B., Portoso, M., Jaschek, R., Tolhuis, B., van Lohuizen, M., Tanay, A. and Cavalli, G. (2009) Functional anatomy of polycomb and trithorax chromatin landscapes in Drosophila embryos. *PLoS biology*, **7**, e13.

10. Thomas, S., Li, X.Y., Sabo, P.J., Sandstrom, R., Thurman, R.E., Canfield, T.K., Giste, E., Fisher, W., Hammonds, A., Celniker, S.E. *et al.* (2011) Dynamic reprogramming of chromatin accessibility during Drosophila embryo development. *Genome biology*, **12**, R43.

11. Li, X.Y., Thomas, S., Sabo, P.J., Eisen, M.B., Stamatoyannopoulos, J.A. and Biggin, M.D. (2011) The role of chromatin accessibility in directing the widespread, overlapping patterns of Drosophila transcription factor binding. *Genome biology*, **12**, R34.

12. Tomancak, P., Beaton, A., Weiszmann, R., Kwan, E., Shu, S., Lewis, S.E., Richards, S., Ashburner, M., Hartenstein, V., Celniker, S.E. *et al.* (2002) Systematic determination of patterns of gene expression during Drosophila embryogenesis. *Genome biology*, **3**, RESEARCH0088.

13. Negre, N., Brown, C.D., Shah, P.K., Kheradpour, P., Morrison, C.A., Henikoff, J.G., Feng, X., Ahmad, K., Russell, S., White, R.A. *et al.* (2010) A comprehensive map of insulator elements for the Drosophila genome. *PLoS genetics*, **6**, e1000814.

14. McQuilton, P., St Pierre, S.E. and Thurmond, J. (2012) FlyBase 101--the basics of navigating FlyBase. *Nucleic acids research*, **40**, D706-714.

15. Gallo, S.M., Gerrard, D.T., Miner, D., Simich, M., Des Soye, B., Bergman, C.M. and Halfon, M.S. (2011) REDfly v3.0: toward a comprehensive database of transcriptional regulatory elements in Drosophila. *Nucleic acids research*, **39**, D118-123.

16. Siepel, A., Bejerano, G., Pedersen, J.S., Hinrichs, A.S., Hou, M., Rosenbloom, K., Clawson, H., Spieth, J., Hillier, L.W., Richards, S. *et al.* (2005) Evolutionarily conserved elements in vertebrate, insect, worm, and yeast genomes. *Genome research*, **15**, 1034-1050.

17. Graveley, B.R., Brooks, A.N., Carlson, J.W., Duff, M.O., Landolin, J.M., Yang, L., Artieri, C.G., van Baren, M.J., Boley, N., Booth, B.W. *et al.* (2011) The developmental transcriptome of Drosophila melanogaster. *Nature*, **471**, 473-479.

18. Cheng, Q., Kazemian, M., Pham, H., Blatti, C., Celniker, S.E., Wolfe, S.A., Brodsky, M.H. and Sinha, S. (2013) Computational identification of diverse mechanisms underlying transcription factor-DNA occupancy. *PLoS genetics*, **9**, e1003571.

**SUPPLEMENTARY NOTES**

**Supp Note 1. Predicting TF Binding Levels in Accessible Regions.** For this, we compared motif scores to ChIP-based binding levels in accessible regions only (SM4). We observed an average PCC of 0.311 (p-value of such a PCC over 2000 points is < 1E-44) in this exercise, and the advantage of multi-species averaging of Stubb scores was no longer observed (Figure 1B). Nevertheless, the high PCC in a large number of data sets, e.g., greater than PCC of 0.3 in 40 of 69 data sets, confirmed that motifs are highly informative of TF-DNA binding levels, even within accessible regions of DNA. Surprisingly, we noted negative PCC values in 8 of the 69 data sets (Table S3), which suggests a gross discordance between biochemical specificities and in vivo occupancy in these cases, similar to some previous reports (6,18). In our evaluations, such discrepancies were observed mostly in ModENCODE data sets, which may in part be because these data sets often correspond to relatively broad developmental intervals and in part due to technical limitations of some of these assays. Excluding the 26 modENCODE data sets, the average PCC in accessible segments, across 43 data sets, increases to 0.45 (p-value < 1E-101).

**Supp Note 2. Fixed Sized TF Target Sets.** TF target sets are defined as the 400 genes with the best maximum TF score in their regulatory regions. We do not claim that these 400 genes are in fact the direct regulatory targets of the TF, or that every TF has the same number of targets. Rather this methodological choice was made in order to ensure parity among the many enrichment tests (one for each TF) that were performed for each expression domain. Different thresholds on the number of genes were tested and did not show improvement on evaluation.

**Supp Note 3. Evaluation of TF-Domain Associations By Expanded Expression**. Of all 5,716 significant TF-domain associations in our compendium, 819 (14%) were supported by the TF gene’s expression in the relevant expression domain (random expectation 5.4%, p-value <1E-158). We note however that for a predicted TF-domain association to be concordant with TF expression data, the TF gene need not be annotated with that expression domain. For instance, repressive TFs are expected to be expressed in spatio-temporal domains bordering the expression domain of their target genes rather than overlapping it. To account for this, we considered a controlled-vocabulary anatomical term hierarchy from FlyBase to identify relationships between expression domains (see SM8), and included 12 types of relationships between domains (Table S6). We now found that 1,232 (22%) of all significant TF-domain associations (Table 1) were supported by the TF’s expression in either the expression domain or a related domain, compared to the chance expectation of 12% (p-value < 1E-97, Figure S7, S8). We also identified all TF-domain pairs where the transcription factor is ubiquitously expressed during the developmental stage of the expression domain. The TF-domain associations that are supported by at least one of these types of TF-expression information, i.e., expressed in the domain, in a related domain or ubiquitously expressed, involved 251 of the 325 TFs that we analyzed (Table S8), and 110 of the 195 expression domains analyzed (Table S9, with domains involving the precursors of ectoderm or epidermis tissue being associated with the most numbers of TFs.

**Supp Note 4. Online Interface.** We have created an online interface for readers to explore the results of our analysis [<http://veda.cs.uiuc.edu/B1H_GRN/>]. The interface divides these detailed results into four major sections; correlation with ChIP data, identification of TF-domain associations, regulatory distance biases, and enhancer models for expression domains. Each section provides a brief description of the types of related results available and the procedure used to generate them. The results are generally contained in sortable tables. At the top of each page is a link back to the home page (“Index”), as well as a link to further details about the table column contents and names (“Glossary”).

**Supp Note 5**. **Alternative Model Evaluation Set.** The “REDfly vs. Enhancers” test set (RFVE) had the same positive examples as the RFVO set (open regions overlapping REDfly enhancers annotated with the domain), but it used a more stringent negative set, comprising open regions that overlapped REDfly enhancers that are annotated with other expression domains. The RFVO test set allows us to evaluate if our classifier distinguishes enhancers of the expression domain from other open regions, while the RFVE test set evaluates our ability to distinguishes enhancers of the expression domain from other validated enhancers of other expression domains. 16 of our 40 linear model classifers exhibited an AUROC of at least 0.7 when evaluated with the RFVE test set, which did not occur in any negative controls)

**Supp Note 6. Identifying Predictive Models.** We use a stricter threshold on performance for models in expression domain that are driven by at least 10 REDfly enhancers. These models are evaluated with our “REDfly vs. Open Regions” (RFVO) AUROCs and “REDfly vs. Enhancers” (RFVE) AUROCs. These performance metrics are over a test set that is composed of positive examples that are validated as affecting expression in the given domain. Because of the high quality positive set, we require that a “good” model have at least 0.7 AUROC on these metrics. However, 155 expression domains lack verified REDfly enhancers to use as positive training and testing examples. We construct a positive set training and testing set from open regions near genes that are expressed in the given domain. These sets are noisy and expected to contain many false positives. The model will be unable to capture these noisy examples and our AUROCs are expected to be lower. For this reason, we use the AUROC threshold of 0.6 on the noisy test set (which is achieved in less than 2% of random controls).

**SUPPLEMENTARY FIGURES**

***Motifs and DNA accessibility together accurately predict genome-wide TF-DNA binding***


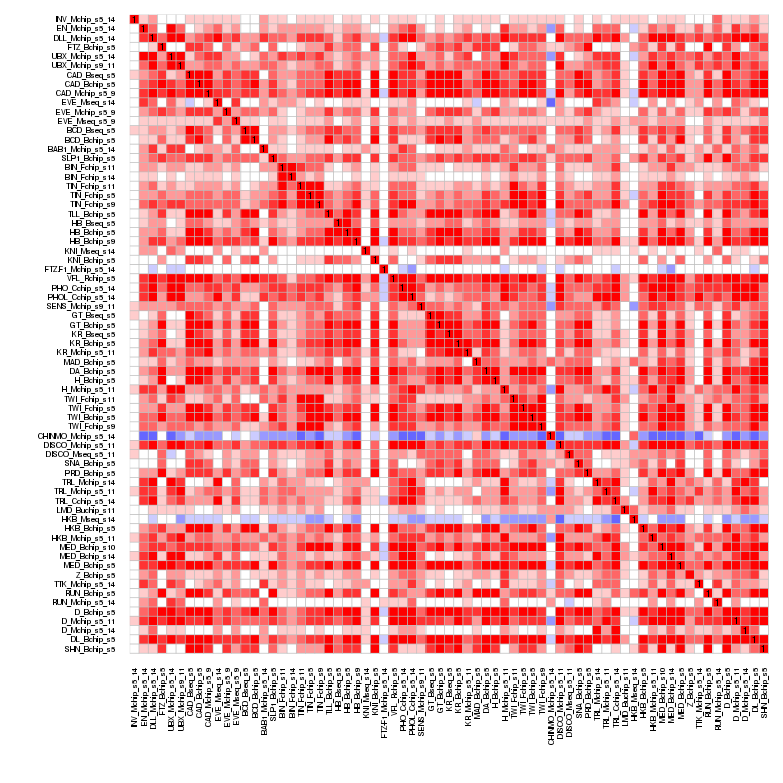


**
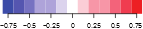
**

**Figure S1. Pairwise correlation of ChIP scores.** The rows and columns are for the 69 ChIP profiles named for the assayed TF, laboratory source, and developmental stage. Each cell is colored for the Pearson correlation between the paired ChIP scores across 1000 non-coding peaks of the row’s ChIP profile and 1000 random regions. Cells on the diagonal are marked with 1s.


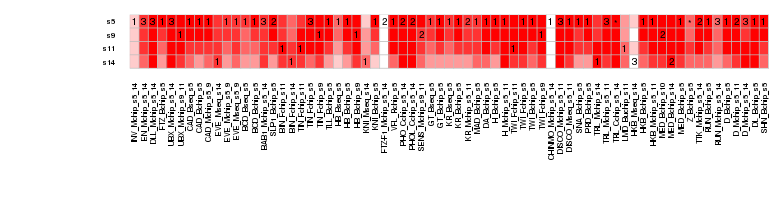


**
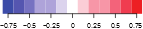
**

**Figure S2**. **Correlation of ChIP scores with chromatin accessibility as measured by DNaseI-seq assays.** The columns of the heatmap represent the 69 ChIP profiles named for the assayed TF, laboratory source, and developmental stage. The rows indicate the DNaseI-seq profiles of chromatin accessibility named for its developmental stage. Each cell is colored for the Pearson correlation between the ChIP score and DNaseI profile scores across 1000 non-coding ChIP peaks and 1000 random regions. For each ChIP track (column), the rank of the matching development stage’s correlation rank of out of 4 accessibility stages is indicated in the appropriate cell.


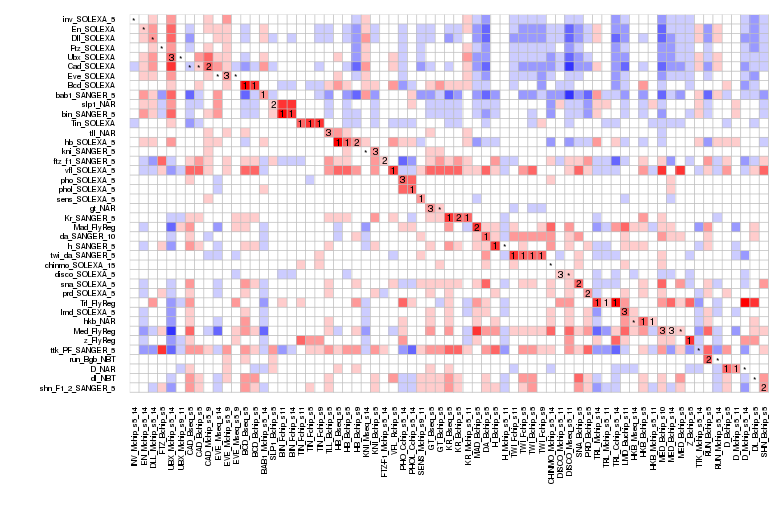


**
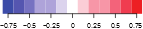
**

**Figure S3. Correlation of ChIP scores with single species motif scores within accessible regions**. Analogous to Figure 1C respectively except single species motif scores were used instead of multi-species motif scores.

***Identification of TFs regulating spatio-temporal expression domains***


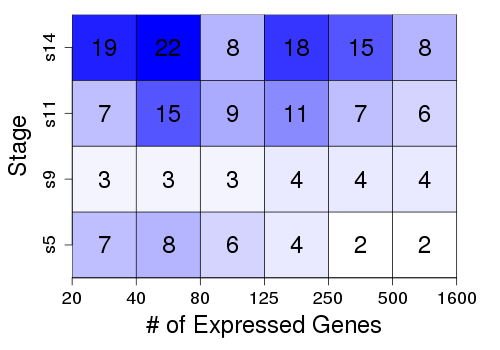


**Figure S4**. **Distribution of expression domain sets by stage and by size.** The 195 expression domains used in this study are from four embryonic developmental time points and contain a subset of the 7,212 annotated genes. The intensity of the cell indicates the number of domains of the given stage and size. Each gene appears in eight expression sets on average with some genes annotated with as many as 60 or more expression domains. Most of the 195 expression gene sets are from the late stage 13-16 and a majority (57%) comprises between 40 and 250 genes


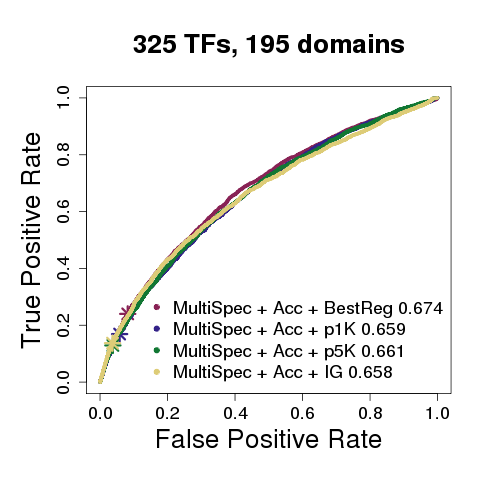


**Figure S5**. **ROC comparison of four different regulatory region methods.** All methods use accessibility filtered, multi-species motif scores, and domain specific expression of the TF as the ground truth. Overall, taking the best regulatory region definition (“BestReg”) has the highest AUROC, but the differences are slight. The starred points represent the significance threshold of 1E-7.

**Figure S6**. **Venn diagram of evidence for 6,825 TF-domain pairs covering 35 TFs and 195 expression domains.** Circles indicate the significant (<1E-7) associations using ChIP scores (yellow), significant (<1E-7) associations using accessibility filtered, multi-species motif scores (red), expression domain specific expression of the TF (green), and related expression domain specific expression of the TF (blue).


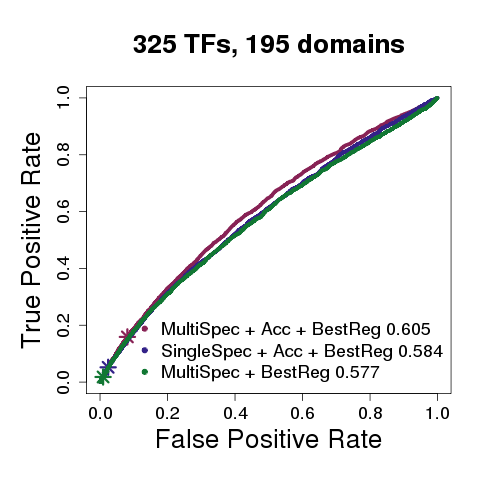


**Figure S7. ROC comparison of methods.** These plots are analogous to Figure 2B with the ground truth including specific expression in a related expression domain that identifies spatially or temporally adjacent expression domains.


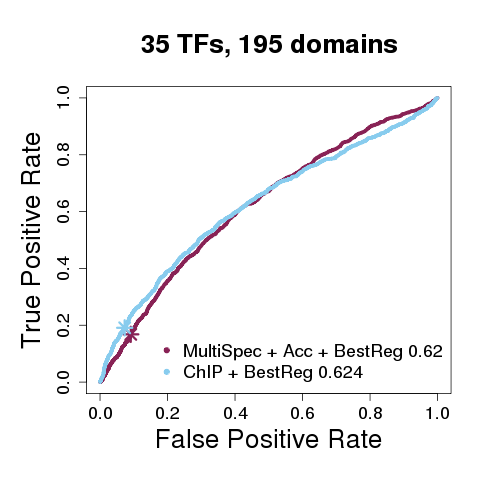


**Figure S8. ROC comparison of methods.** These plots are analogous to Figure 2C with the ground truth including specific expression in a related expression domain that identifies spatially or temporally adjacent expression domains.

**Figure S9. Clypeolabrum network example**. Same as Figure 3A except included additional edges from TFs with redundant motifs. The lighter colored edges to a transcription factor mean that there is a TF with a similar motif that is significantly associated with more specific expression support or that has the same type of expression support but is more significantly associated (see SM9).


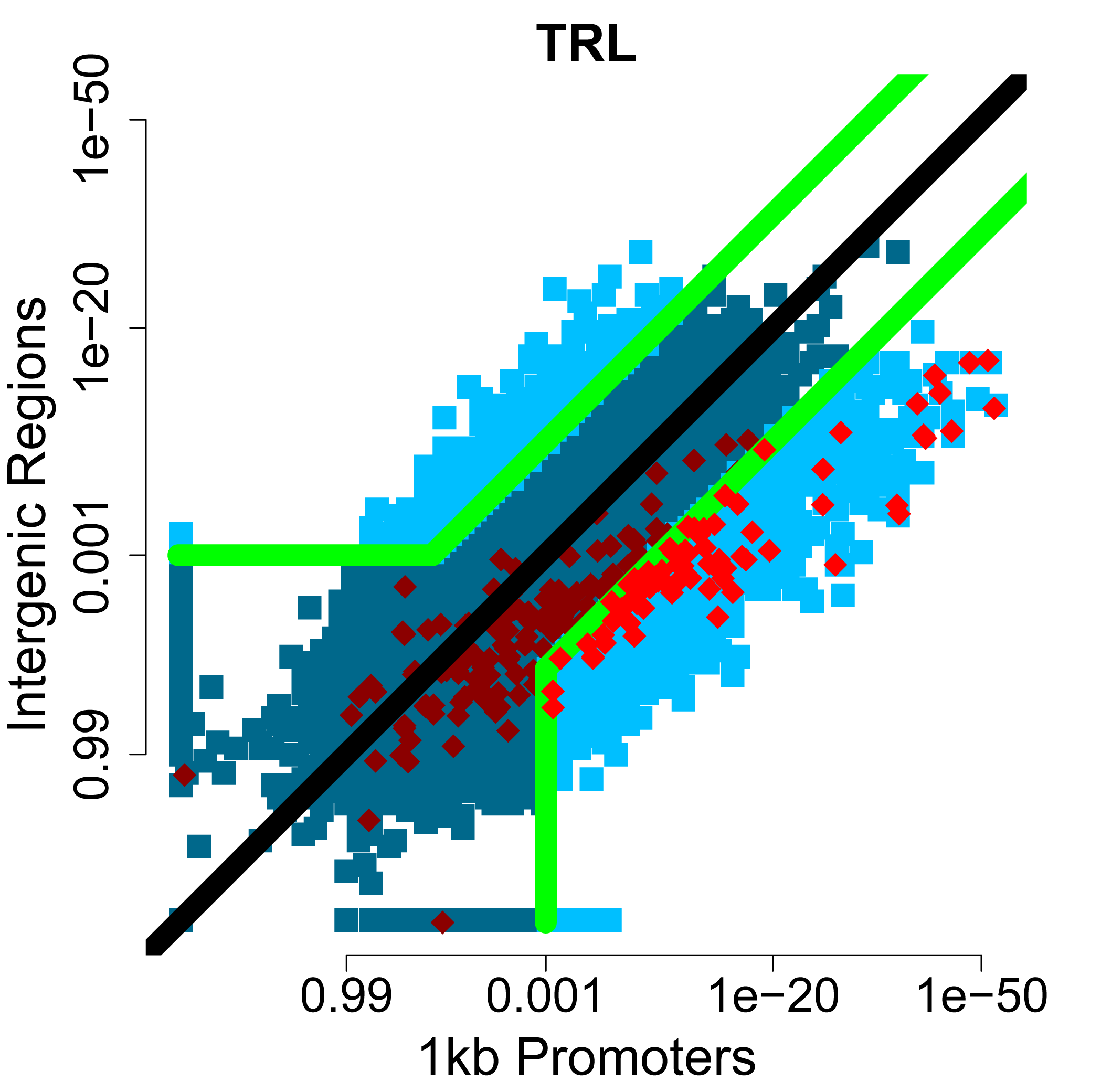


**Figure S10. TRL is biased for proximal “p1K” regulation.** The plot shows the p-values of 325*195 TF-domain association tests using the “p1K” (“IG”) regulatory region in the x-axis (y-axis) in blue. The red points indicate all of the association results involving TRL. The diagonal (black line) is shown as well as the difference required between the two p-values to be considered a bias (green lines)

***Enhancers associated with expression domains***


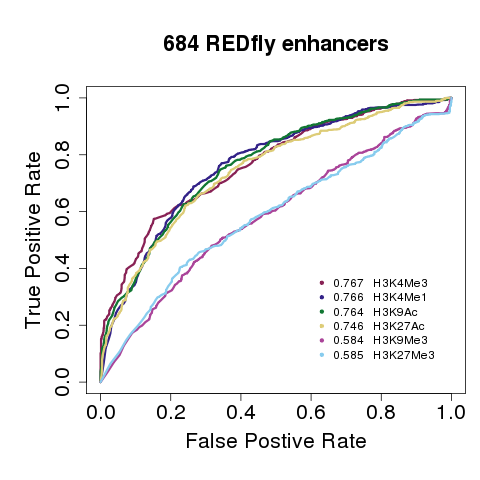


**Figure S11. ROCs for methods of detecting 684 REDfly enhancers from 684 negative sequences**. In the legend, the AUROC is reported for each curve from the particular data type. The figure shows the chromatin marks H3K4Me3, H3K4Me1, H3K9Ac, and H3K27Ac are much better than the marks H3K9Me3 and H3K27Me3.


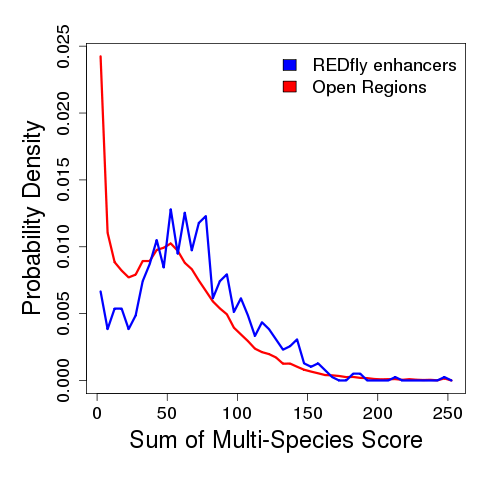


**Figure S12. Distribution of the sum of multi-species motif scores. Each** open region is has non-negative multi-species z-scores for 325 motifs which can be summed. The distribution of this sum over all open regions that overlap REDfly enhancers is shown in blue, while the distribution of the sum over all open regions is shown in red. We removed open regions with a sum less than 10 because these are least consistent with the REDfly distribution.

**
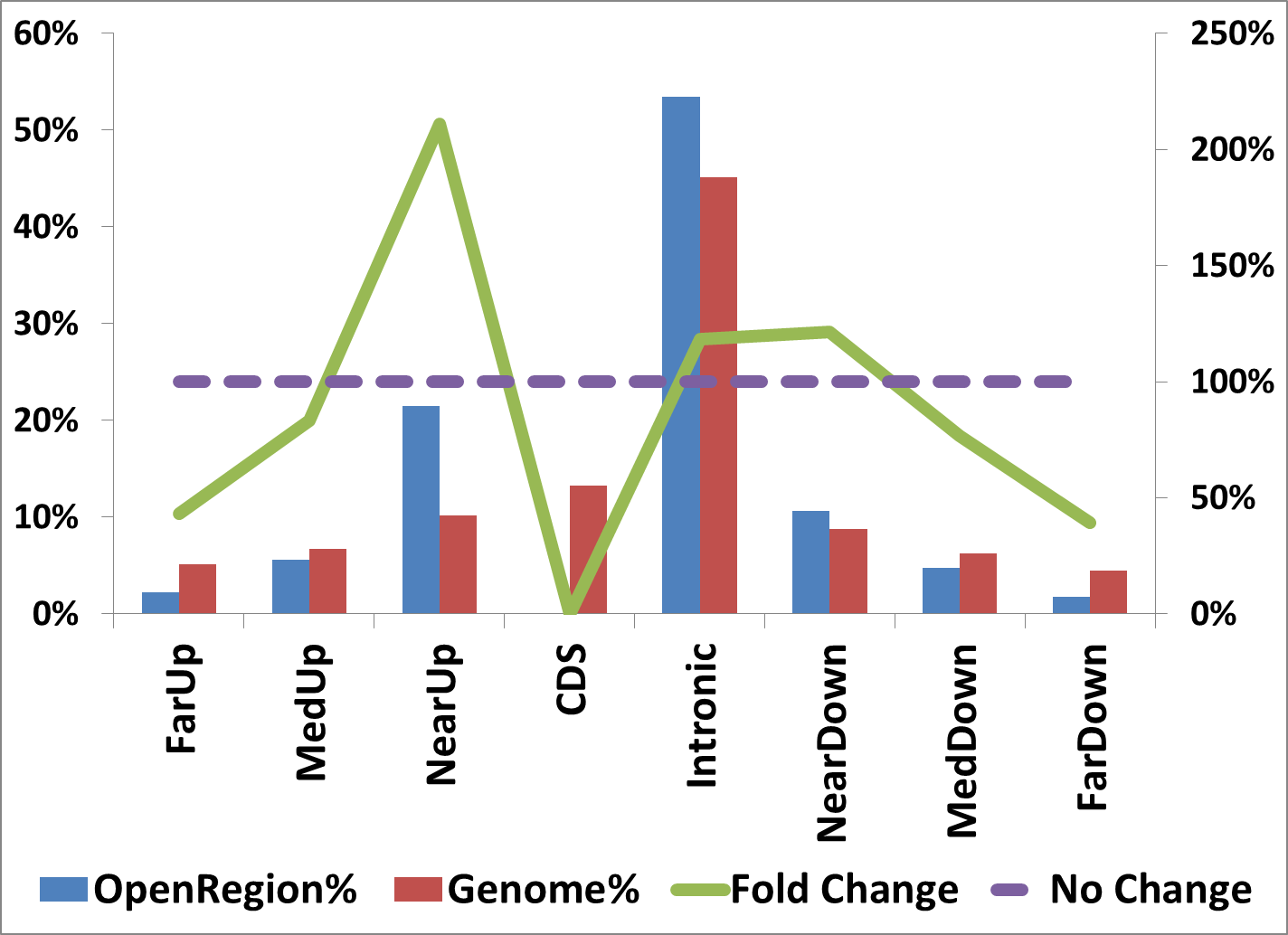
**

**Figure S13. Relative genomic location of open regions**. Regions are assigned a label based on their position relative to their nearest gene depending on whether they are intronic, exonic (CDS), or >20kb (Far), 5-20kb (Med), <5kb (Near) from the upstream or downstream end of the gene. The distribution of the open regions (blue bars) is compared to the genome wide distribution (red bars) on the left axis, with their fold change plotted in green on the right axis.


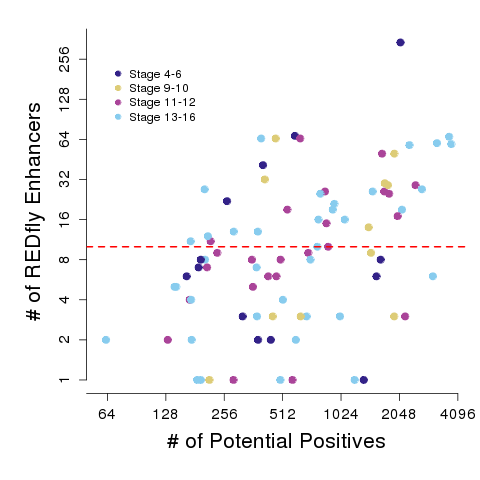


**Figure S14. Count of enhancers by expression domain**. Each point represents an expression domain and is colored according to the developmental stage of the domain. The x-axis represents the number of open regions that are accessible in the developmental stage and that are within 5kb of a neighboring gene annotated with the expression domain. These represent the pool of positive training examples for each domain. The y-axis represents the number of open regions for each expression domain that overlap REDfly enhancers annotated with the domain. The 40 expression domains above the red dotted are evaluated with the REDfly based test sets (RFVO and RFVE).


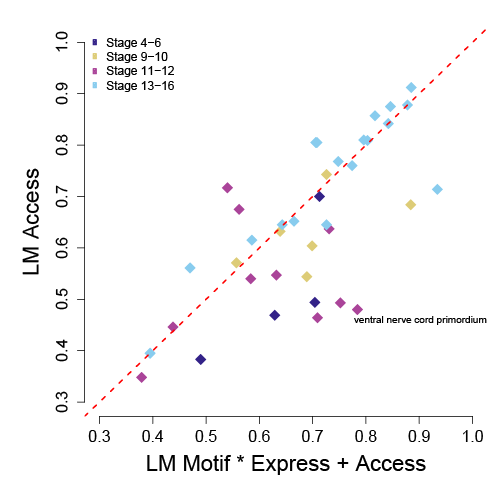


**Figure S15. Comparison of the RFVO AUROCs.** One point is plotted for each of the 40 expression domains with the color indicating its developmental stage. The x-axis (y-axis) is the AUROC of the “Motif * Express + Access” (“Access”) model. Off diagonal points (labeled) are expression domains that find better models in using one set of features instead of the other. The expression domains that seem to benefit most using motif features over ChIP-based ones are enriched in early stages of development.

| 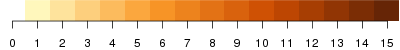 | | |
| --- | --- | --- |
| **Test** | **RFVO** | **RFVE** |
| 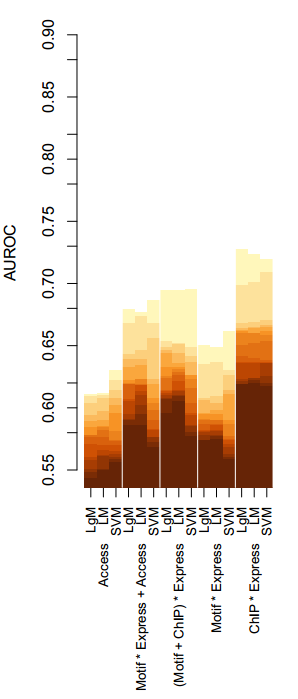 | 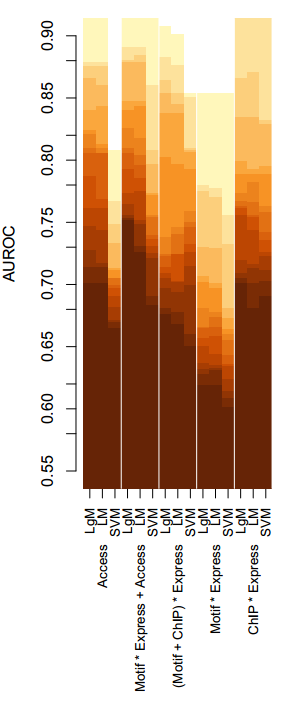 | 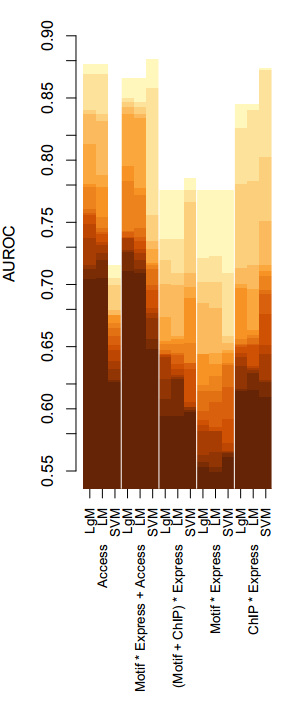 |

**Figure S16. AUROC comparisons for 40 expression domains using several different models.** For each of the 40 expression domains, we form three different test sets, the standard (Test), REDfly vs. Open Regions (RFVO), and REDfly vs. Enhancers (RFVE) (see SM13 and Supp. Note 5). For a given model and test set, we calculate the AUROC for each of 40 expression domains using that model and test set. The distribution of these 40 values is visualized as a column in the figure with the intensity of the column indicating the number of expression domains that have an AUROC at or above the y-axis value. This figure compares across several models. The three types of classifiers used were linear models (“LM”), logistic regression models (“LgM”), and support vector machines (“SVM”). The features in the model either combined 325 motif based features with four accessibility based features (“Motif * Express + Access”), only combined the 325 motif based features (“Motif * Express”), only combined the 4 accessibility features (“Access”), combined the 69 ChIP-based features (“ChIP * Express”), or combined the 325 motif based and 69 ChIP-based features ((Motif + ChIP) * Express).

**
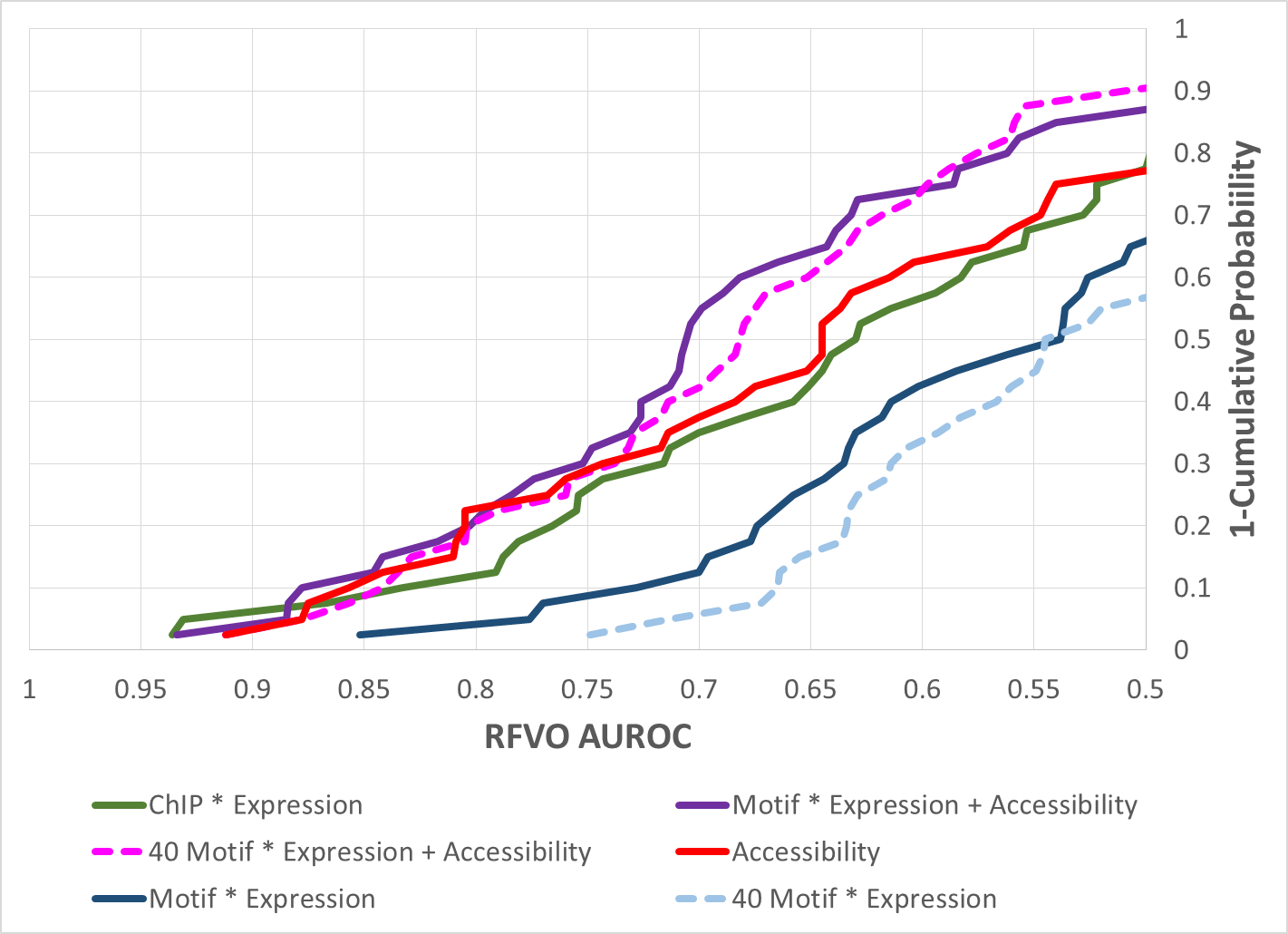
**

**Figure S17. Comparisons of six different models**. The results from Figure 4B are recreated here (solid lines) with two additional models (dotted lines). These new models (“40 Motif * Expression” and “40 Motif * Expression + Accessibility”) use only motifs for the 40 transcription factors that had ChIP data used in the “ChIP * Expression” (SM14) model. As expected, the “40 Motif * Expression” model does worse than the corresponding “Motif * Expression” which has motif scores for all 325 TF motifs. Interestingly, the “40 Motif * Expression + Accessibility” model outperforms the ChIP based model even though it uses only motifs from ChIP’ed TFs. The complete model “Motif * Expression + Accessibility” with all 325 motifs for mostly outperforms this model restricted to 40 motifs.


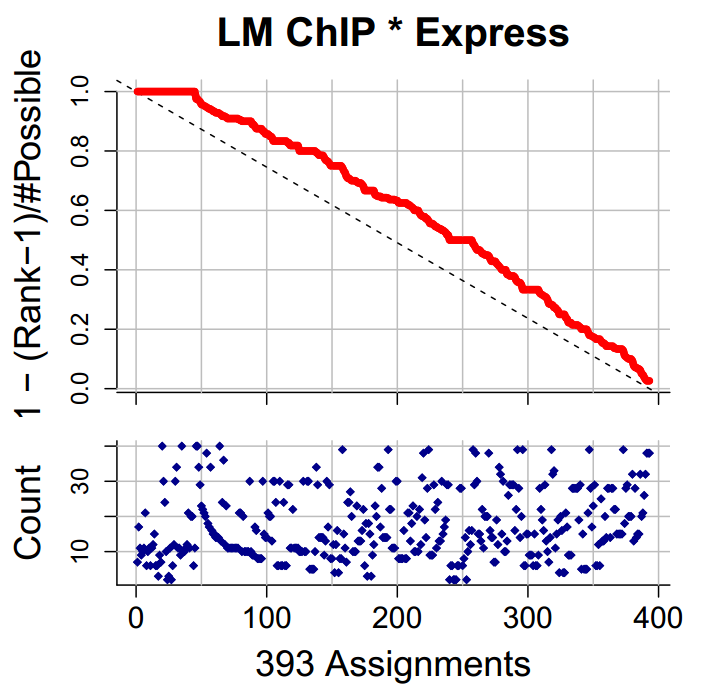


**Figure S18. Assignment evaluation of method.** 393 open regions (x-axis) overlap REDfly enhancers. Each of these open regions has a number of possible expression domains that it can be assigned (lower plot, blue dots). For each open region, we order the possible expression domains by the predictions of our “ChIP * Express” models and identify the rank of the expression domain annotated for the enhancer in REDfly. We plot a non-negative statistic (upper plot, red line) that achieves a maximum possible value of 1 when the REDfly domain is the best of all possible expression domains of that open region.

**SUPPLEMENTARY TABLE LEGENDS**

**Table S1. 69 ChIP Profiles.** Lists the 69 ChIP profiles we downloaded by the profile name, transcription factor, laboratory source, experimental type, developmental stages, and related FlyFactorSurvey motif. 53 profiles with “Eval” equal to 1 are used to evaluate TF-expression associations.

**Table S2. 325 Motifs.** Lists the 325 motifs used in this study by the FlyFactorSurvey name, the family of their DNA binding domain, and the common name of their corresponding transcription factor.

**Table S3. Comparison of Model Scores Correlated with ChIP.** Columns are the seven model types and rows are the 69 transcription factors. Cell values are the Pearson correlations between model scores and ChIP scores for 2000 windows. Row and column are sorted by their calculated averages. The final column indicates with a 1 the ChIP profiles from ModENCODE, which fall disproportionately at the bottom of the list.

**Table S4. Multi-species Motif Scores Correlated with Accessibility.** Columns represent four different developmental stages and rows are 40 selected transcription factor motifs. Cell values are the Spearman correlations between multi-species motif scores and accessibility scores for 2000 windows. The rows are sorted by their calculated averages, which appears in the final column.

**Table S5. 195 Expression Domains.** Lists the 195 expression domains by our term identification number (TID), the developmental stage, the BDGP expression domain annotation name, the number of genes that are labeled with that expression domain (#ExpGenes), and the corresponding anatomical term in the FlyBase term hierarchy (“-” if not match was found).

**Table S6. Summary of Term Pairs by Relationship Type.** Lists the 12 different types of relationships used in defining the related expression domains and the number of pairs of BDGP terms that were found to have that relationship.

**Table S7. 1068 Term Relationships Extracted from FlyBase.** Each expression domain term in the first column (or one of its synonyms) was found to have the second column’s relationship to the expression domain term in the third column (or one of its synonyms).

**Table S8. Summary of Significant Associations by Motif.** For each motif, lists the number of 195 possible expression domains that were significantly associated at 1E-7 “Signif Assoc”. Of these, shows how many had specific expression support “ED Spec Exp”, specific support in a related domain “Rel ED Spec Exp”, or stage-specific ubiquitous expression support “Stage Spec Ubiq”.

**Table S9. Summary of Significant Associations by Expression Domain.** For each domain, lists the number of 325 possible motifs that were significantly associated at 1E-7 “Signif Assoc”. Of these, shows how many had specific expression support “ED Spec Exp”, specific support in a related domain “Rel ED Spec Exp”, or stage-specific ubiquitous expression support “Stage Spec Ubiq”.

**Table S10. Top 25 Significant Associations Supported by Phenotypic Data Curated by Flybase**. Each row represents an association between a TF motif and an expression domain of a particular stage. The significance of the association is reported as a negative log p-value “nlog_pval”. These associations all have expression support, either with the TF expressed in the specific “ED Spec Exp” or a related domain “Rel ED Spec Exp”. Additionally, these associations are supported by the curated FlyBase database of phenotypes associated with mutant alleles. REDfly lists the “FlyBase Expression Domain” as a phenotype for fly mutants involving the motif TF.

**Table S11. Summary of Regulatory Region Preference.** Percentage of all significant motif associations “Signif Assoc”, all significant associations with specific expression support “ED Spec Exp”, all significant associations with specific expression in related expression domains “Rel ED Spec Exp”, all significant associations with stage specific ubiquitous expression “Stage Spec Ubiq”, and all significant associations without expression support “Unsupported” by the regulatory region of the greatest association significance.

**Table S12. Motif Regulatory Region Preference.** For each motif, lists its DNA binding domain family, the number of significantly associated ( < 1E-7) expression domains out of 195, “sig”, as well as those that significantly more significant with “p1K” regulatory region, “sig_p1K”, or those that are significantly more significant with “IG” regulatory regions, “sig_IG”. The final column is the negative log one-sided Fischer exact test p-values of enrichment of the significant “p1K” or “IG” specific TF-domain pairs with the TF-domain pairs of “sig”. Positive values are the enrichment for “p1K” and negative values are for “IG”.

**Table S13. Expression Domain Regulatory Region Preference.** For each expression domain, lists the number of significantly associated ( < 1E-7) motifs out of 325, “sig”, as well as those that significantly more significant with “p1K” regulatory region, “sig_p1K”, or those that are significantly more significant with “IG” regulatory regions, “sig_IG”. The final column is the negative log one-sided Fischer exact test p-values of enrichment of the significant “p1K” or “IG” specific TF-domain pairs with the TF-domain pairs of “sig”. Positive values are the enrichment for “p1K” and negative values are for “IG”.

**Table S14. Distribution of Open Regions.** Regions are assigned a label based on their position relative to their nearest gene depending on whether they are intronic, exonic (within CDS), or >20kb (Far), 5-20kb (Med), <5kb (Near) from the upstream or downstream end of the gene. The distribution of the open regions (in first column) is compared to the genome wide distribution (in second column) with their fold change (in third column).

**Table S15. Evaluations of the “LM Motif * Express + Access”.** For each of 195 expression domains, lists the number open regions that serve as positive examples (“Positive Regions”), and the number of those examples used as positives in training (“Training Set Positives”) and testing (“Testing Set Positives”). “RF Testing Set Positives” is the number of open regions that overlap REDfly enhancers annotated with the given domain. The three “AUC” columns show the AUROC for the expression domain model for each of the three possible test sets defined in SM13 and Supp Note 5. A value of 0 means that the AUROC was not computed. Yellow highlight indicates an AUROC value that is strong enough to trust the model in prediction. “Non-zero Features” indicate how many of the 325 motif plus 4 accessibility features are used in training because they show variation across the examples. “Signif Features” indicate how many of those features are determined to be statistically significant (p-value < 0.01). Finally, the “Best Features” list the top 3 most significant features of each expression domain model.
